# Supplementary material for: Gray whale (Eschrichtius robustus) post-mortem findings from December 2018 through 2021 during the Unusual Mortality Event in the Eastern North Pacific
Source: PLoS One. 2024 Mar 27;19(3):e0295861. doi: 10.1371/journal.pone.0295861 (PMC10971505; doi:10.1371/journal.pone.0295861)
Supplement: S1 Appendix — Gray Whale UME Dead Whale Nutritional Condition Protocol & Table. (DOCX) [file pone.0295861.s001.docx]

**S1. Gray Whale UME Dead Whale Nutritional Condition Protocol & Table**

**Purpose:**

To standardize photographs, external and internal examinations, and descriptions to assess nutritional body condition of stranded gray whales.

**Plan:**

To use data from all stranded live, fresh dead, and moderately decomposed gray whales using the below protocol and table to categorize whales into the following nutritional condition categories:

- 1 Emaciated (Poor Nutritional Condition);
- 2 Thin (Fair Nutritional Condition);
- 3 Average (Moderate Nutritional Condition); and
- 4 Fat (Good/Excellent Nutritional Condition)

**Process – External Evaluation/How to Take Photos:**

BEFORE touching the whale

- Photos taken from beside the whale, lateral (right angle to whale) views of:
  - Thoracic and lumbar epaxial regions to demonstrate vertebral process prominence ( or lack of)
  - Post nuchal area (“peanut head site”)
- Photos taken from an oblique angle of the whole body facing towards head and towards fluke to demonstrate scapula area and vertebral process prominence;
- Photos of the genital slit area from the umbilicus (level with distal tip of pectoral flipper) to anal sac region or mid-peduncle;
- Photo-id protocol of markings (see existing protocol); and
- If ventral side up – photos taken at an oblique angle to show the pectoral and abdominal muscles, mandibles.

**Process – Internal/Necropsy Evaluation:**

- Examine and document blubber characteristics, presence and consistency of subcutaneous fat, and status of internal fat stores (nuchal pad, mesenteric, omental, peri-renal, mediastinal, coronary groove; see protocol below).
- Collect blubber for Histo - full depth histo at lateral axillary and optionally at max girth sites, cut blubber into three pieces– label superficial, middle, deep prior to putting in formalin

**Scoring:**

External (n=4) and Internal Features (n=4) are scored on a scale of 1 (emaciated) to 4 (fat) or assigned “CBD” (could not be determined) or “NE” (not examined). External features include nuchal fat pad profile, vertebral process prominence, epaxial muscle profile, and scapular prominence (live whales only). Internal features include blubber oiliness, blubber texture, subcutaneous fat, and visceral fat (Table A1). The final score is the most common score between all scored features (see Table A2). If features are categorized evenly between two nutritional categories, the higher score takes precedence ( e.g. two features with a score of 1 and two features with a score of 2, result in a final score of 2.

Table A1. Nutritional Body Condition Status Score Table

|  |  | **Nutritional Body Condition Status Score Table** | | | |
| --- | --- | --- | --- | --- | --- |
| **#** | **Feature-**  **External Exam** | **1 Emaciated (Poor)** | **2 Thin (Fair)** | **3 Average (Moderate)** | **4 Fat (Good/Excellent)** |
| 1 | Nuchal fat pad area | Marked dipping  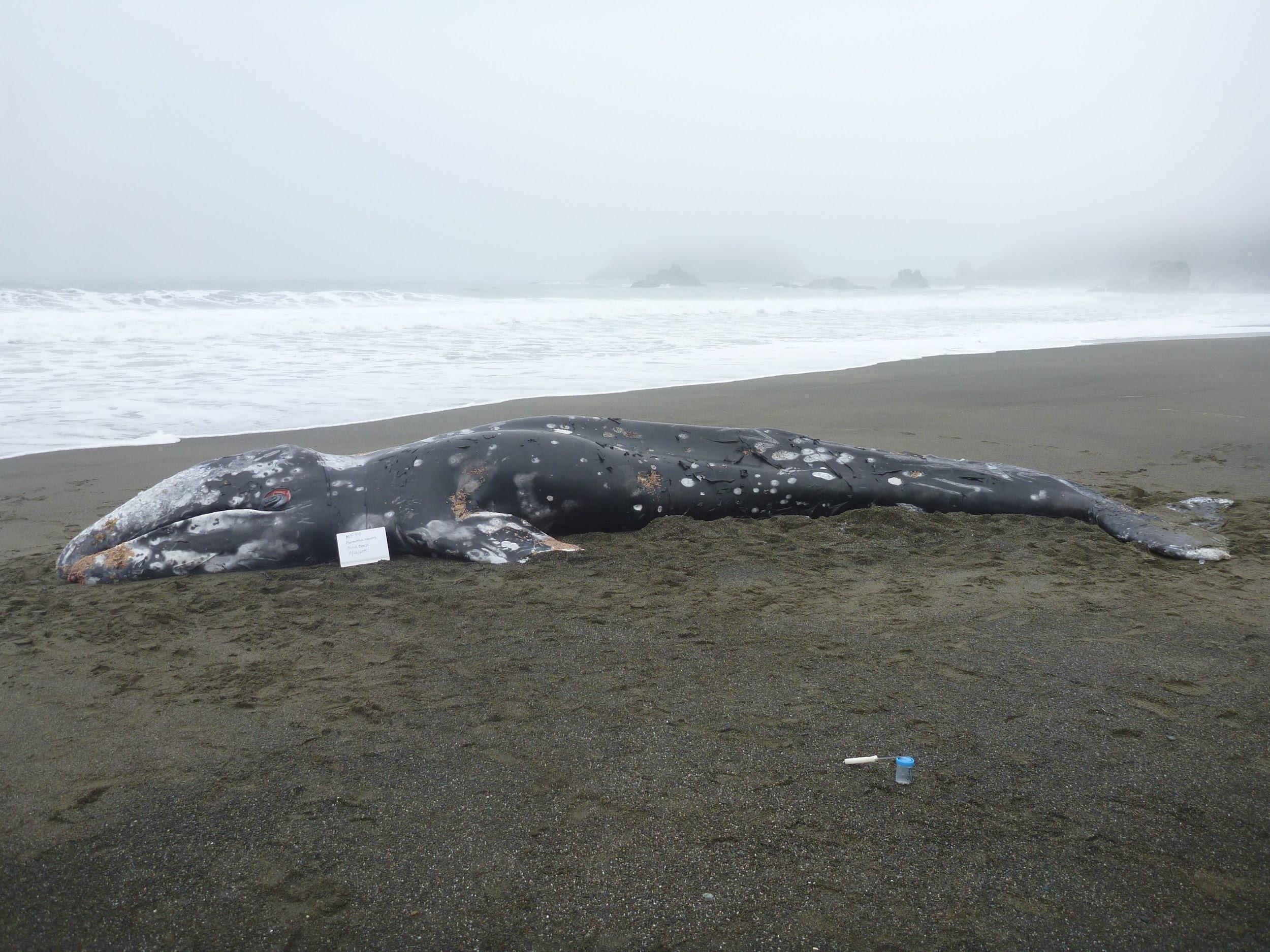 | Slight dip  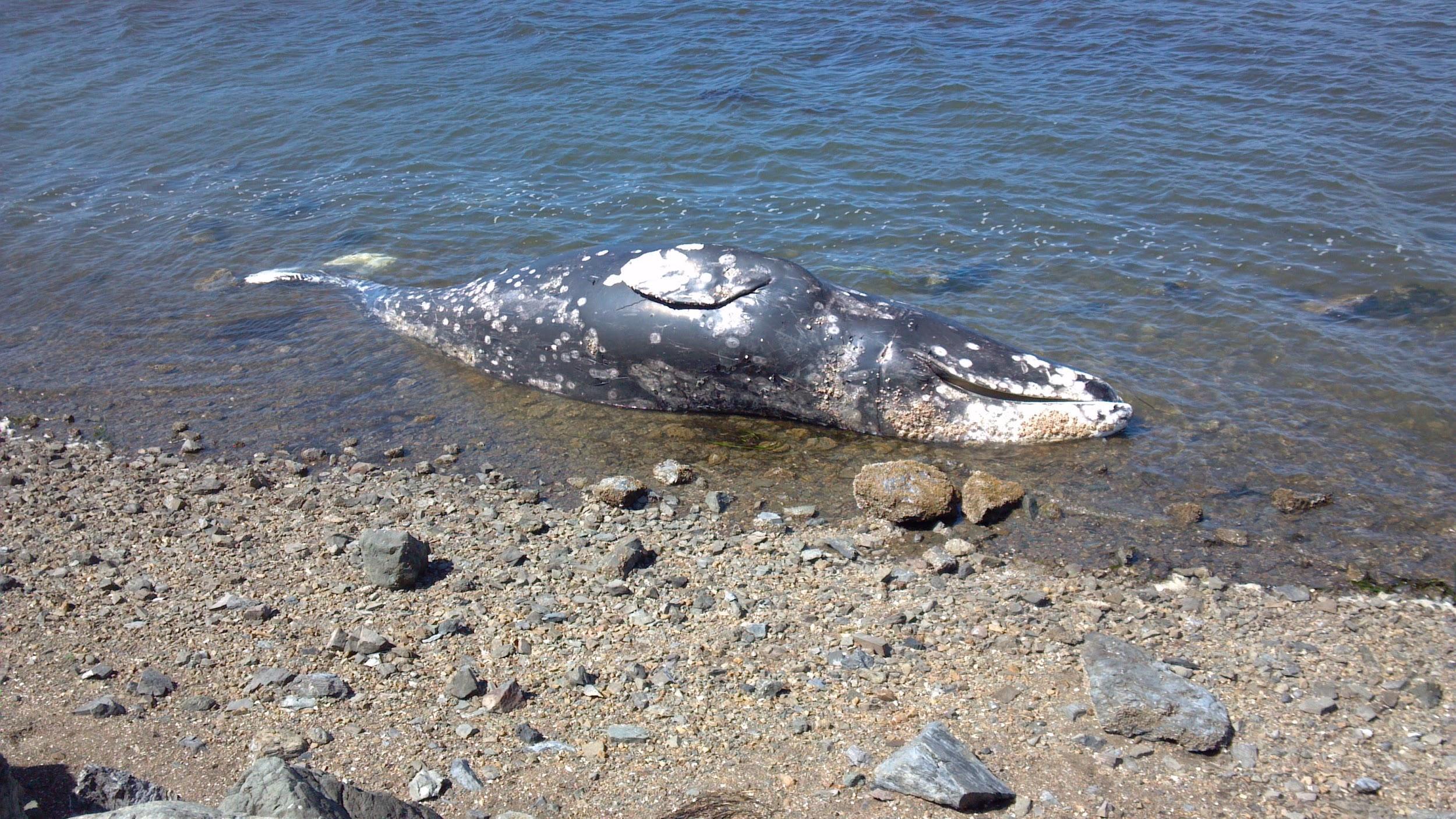  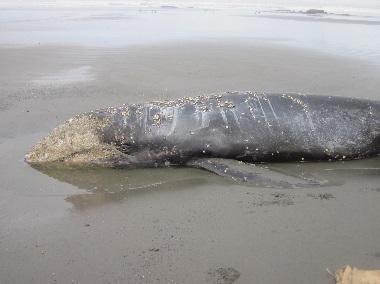 | Flat  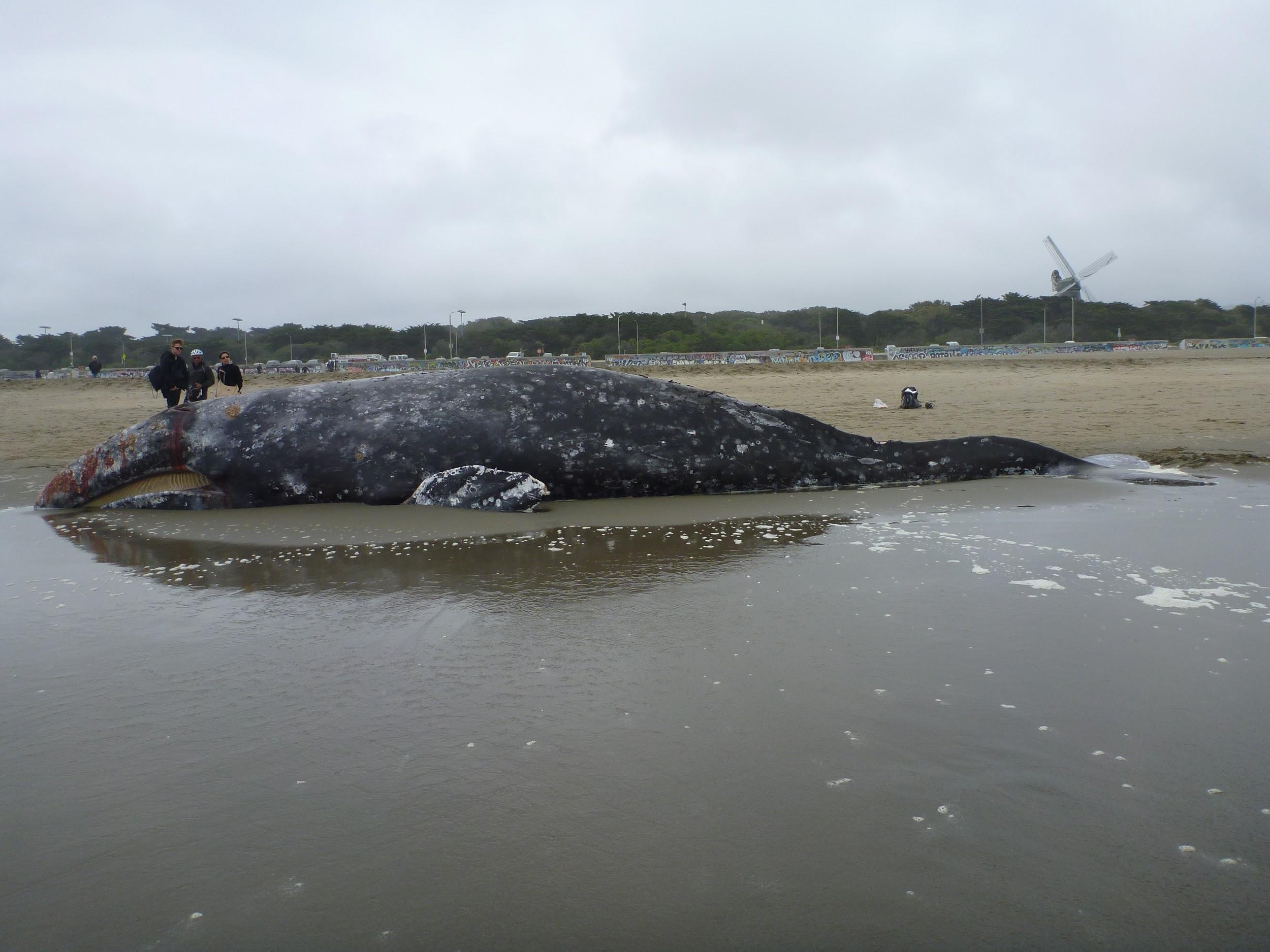 | Convex  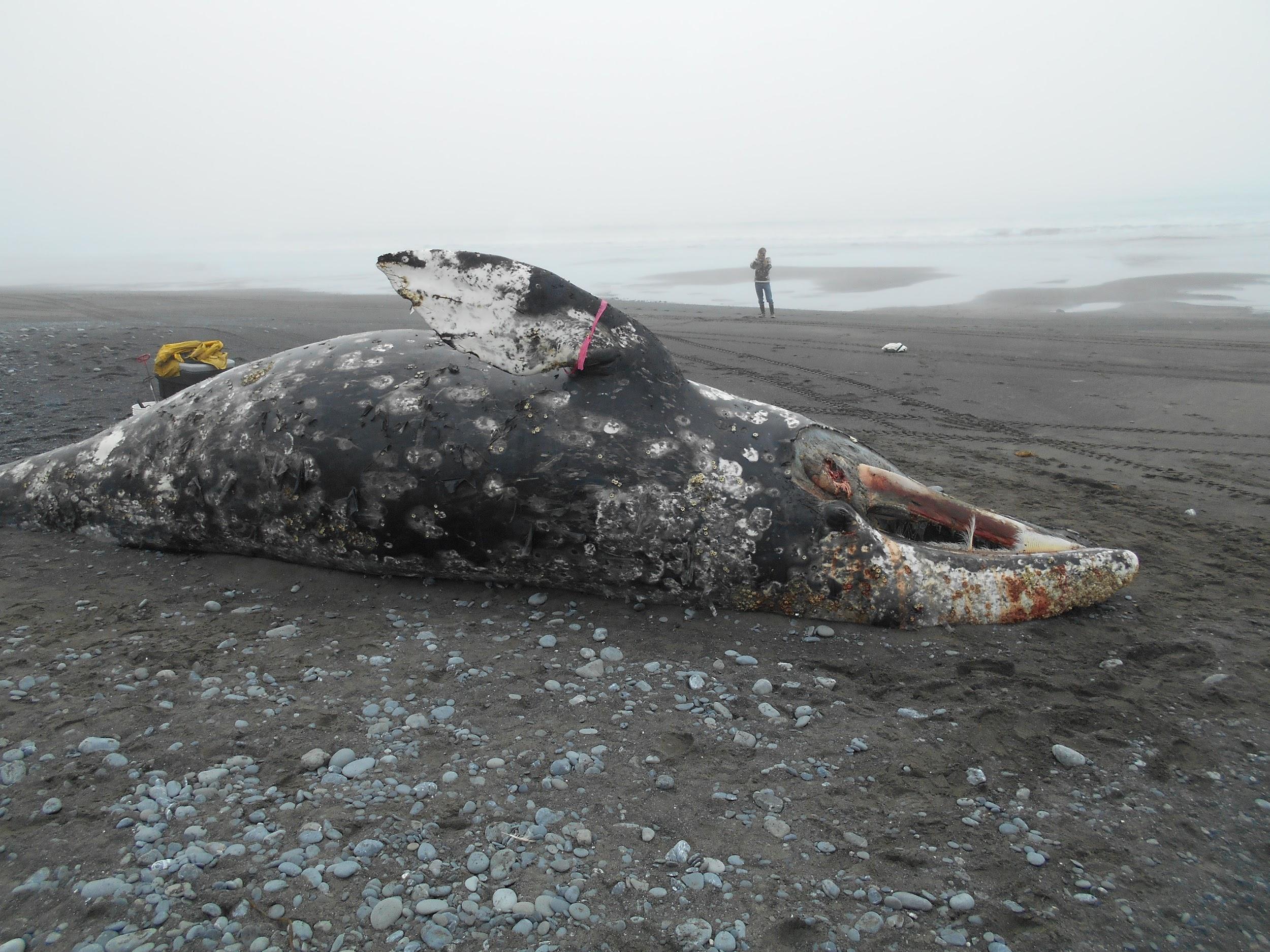 |
| 2 | Vertebral processes | Prominent processes  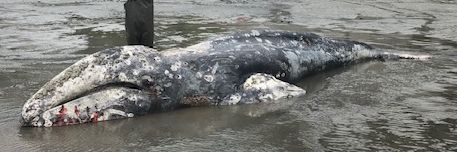 | Shape detectable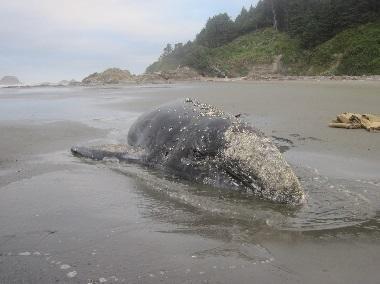 | Not visible | Very rounded profile |
| 3 | Epaxial muscle profile (thoracic and lumbar views) | Sharply concave musculature  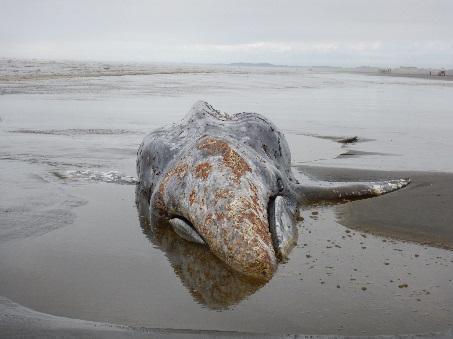 | Slight dipping of the epaxial areas  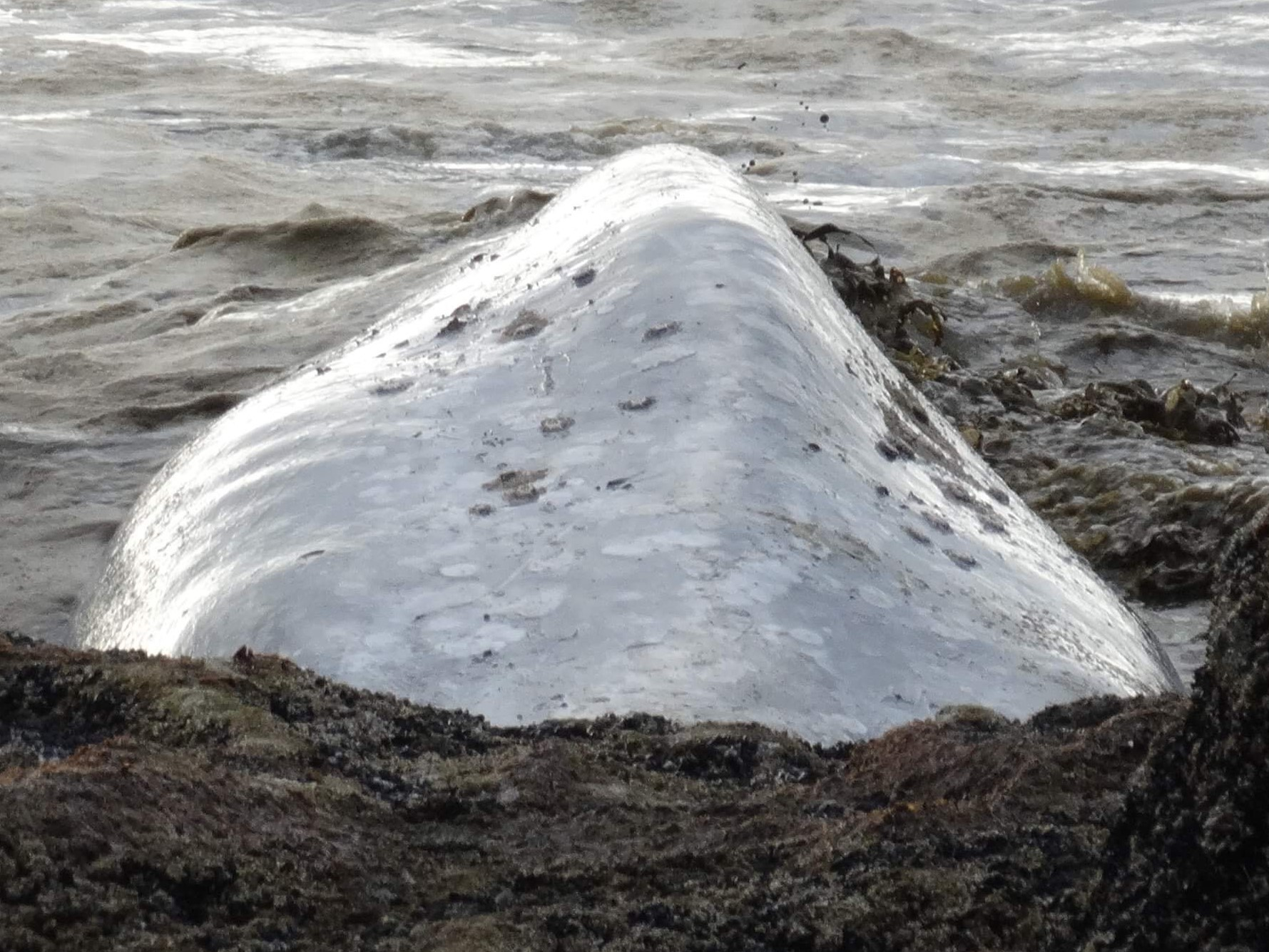 | Flat | Rounded convex (add new photo)  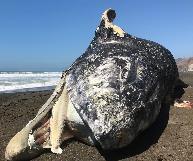 |
| 4 | Scapulae prominence **(Live whales only)** | Very visible | Just visible | Not visible | Not visible (see nuchal fat photo above) |
| # | **Feature-**  **Internal Exam** | **1 Emaciated (Poor)** | **2 Thin (Fair)** | **3 Average (Moderate)** | **4 Fat (Good/Excellent)** |
| 5 | Blubber Characteristics - *Blubber Oiliness*  *note that very decomposed whales will seep oil, making it appear more oily than expected | Watery or Dry  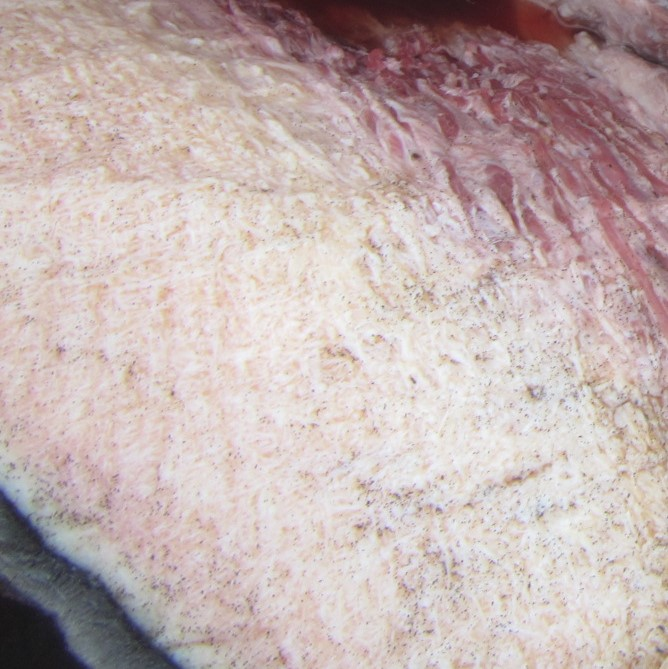 | Low oil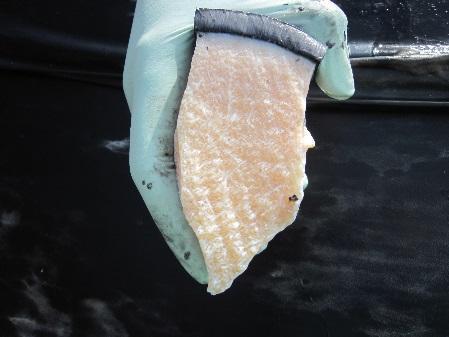 | Moderately oil (can see/feel oil but not dripping) | Very oily, oil drips when cut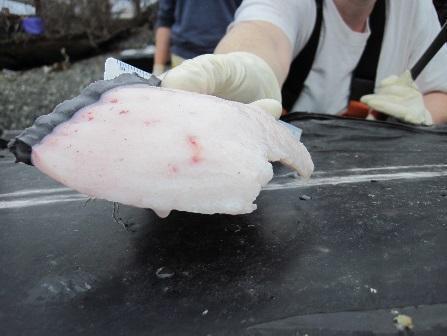 |
|  | *Blubber Texture*  *note that decomposition can make blubber more pliable | Very fibrous | Moderately fibrous | Somewhat to moderately pliable | Very pliable |
| 6 | Subcutaneous fat | None  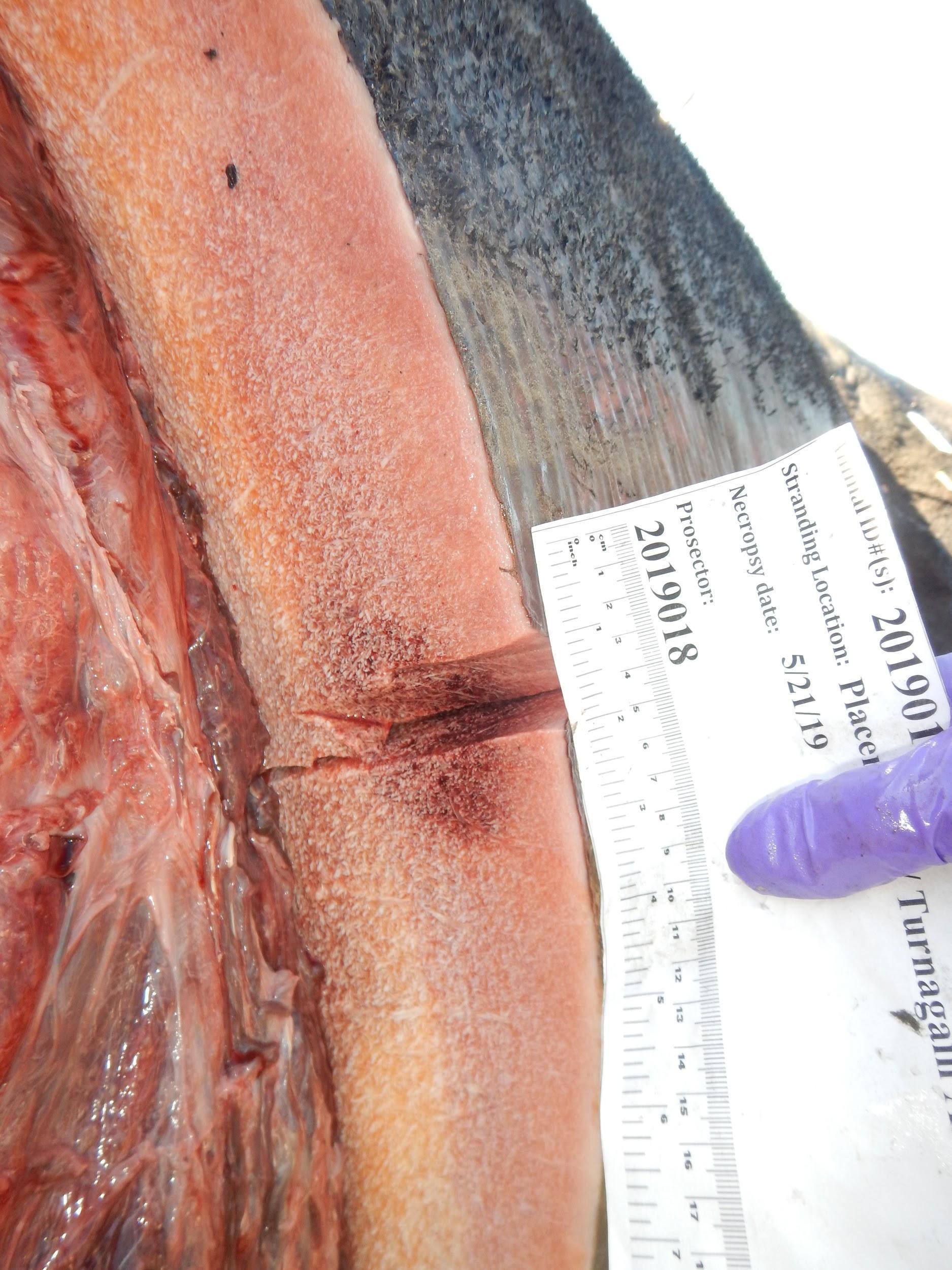 | Scant | Moderate | Abundant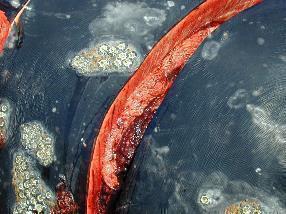 |
| 7 | Fat in coronary groove, around kidneys, in mesentery, omental and mediastinum | None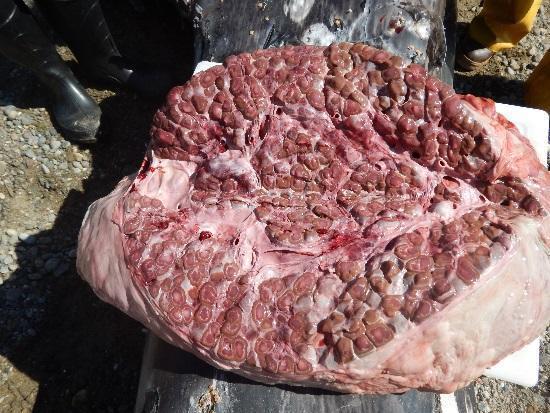 | Scant | Moderate  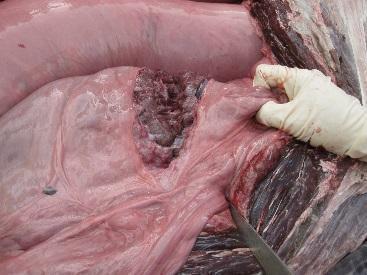 | Abundant |
|  | **Other Features (Supportive):** |  | | | |
|  | Serous Atrophy of Fat | Yes/no?: Describe anatomic location  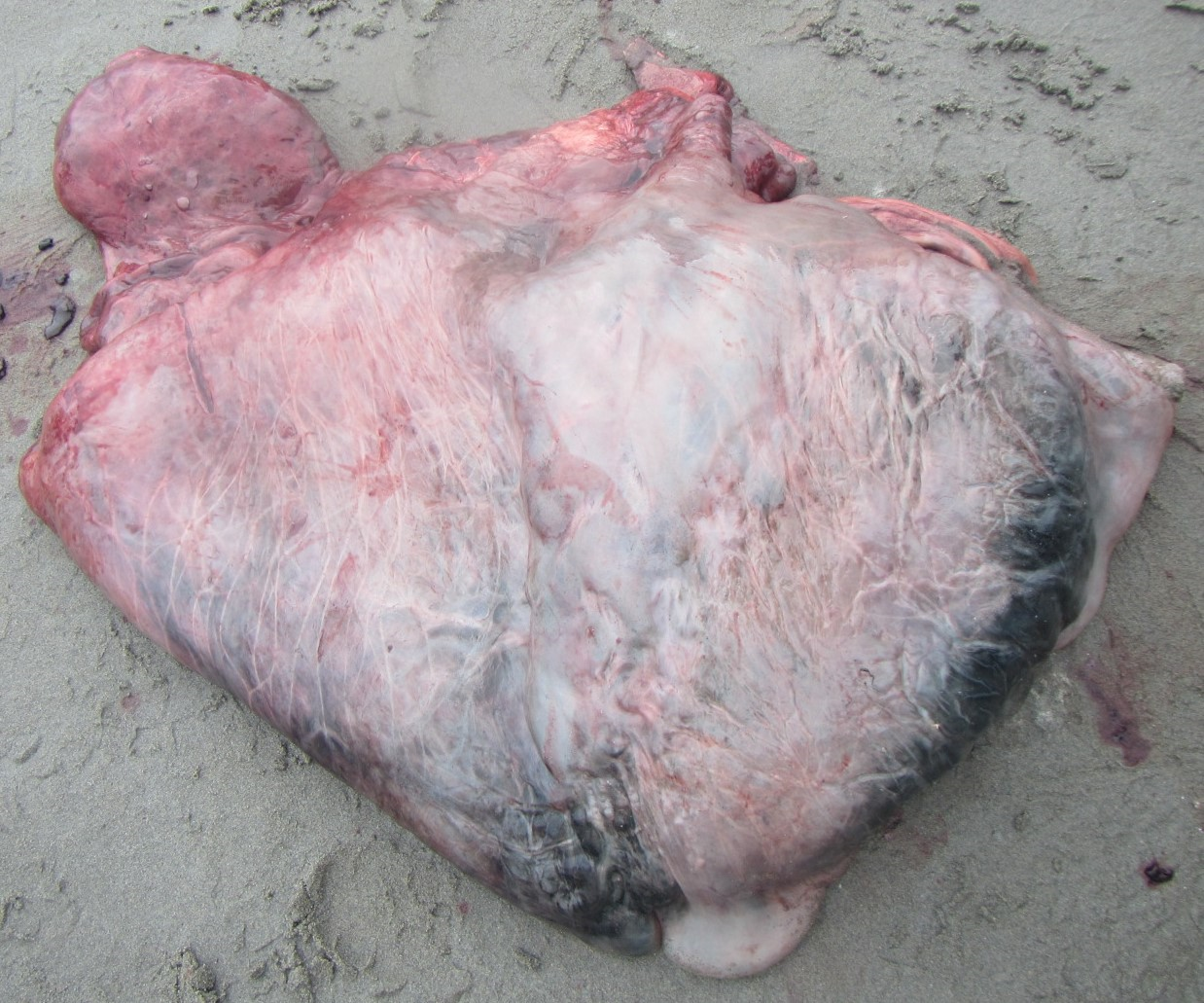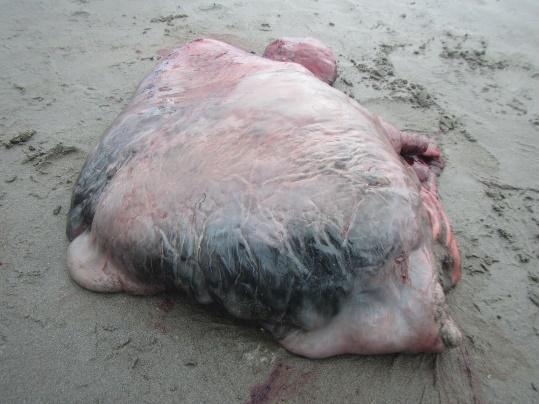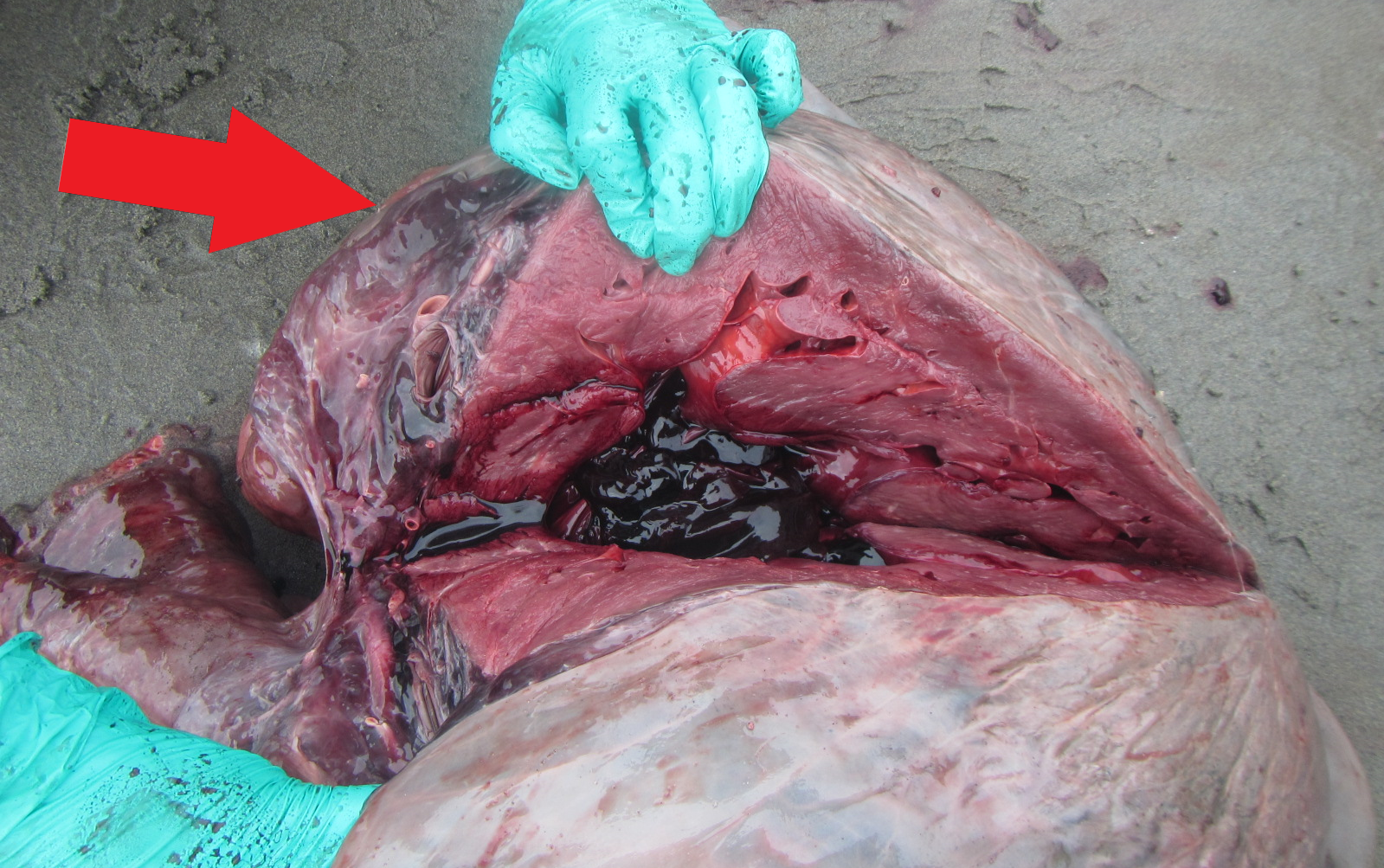 | | | |
|  | Blubber Color? | Yes/no?: Note color - Pink; Orange/Salmon; Yellow; Cream/Tan; White | | | |
|  | Blubber Red-Band? | Yes/no?: Describe – Red band inside layer; Red band mid-layer; Red band outer layer; None/Color even throughout | | | |
|  | Cyamid load? | Yes/no?: Describe Location and Percent coverage (note which species of cyamids) | | | |
|  | Gastrointestinal Contents? | Yes/no?: Describe (including gas) | | | |
|  | Gastrointestinal Parasites? | Yes/no?: Describe and location | | | |
|  | Lipid Data? | Yes/no?: Include lipid% and triglyceride % (if available) | | | |
|  | Histologic Nutritional Information | If histopathology tissues are sampled include: Presence/absence, extent (superficial, middle or deep layer), and severity (moderate, marked, severe) of fat atrophy, muscle atrophy, hemosiderosis | | | |
|  | Overall Body Shape | Snake-like, full body elongated fusiform appearance. Yes/no?  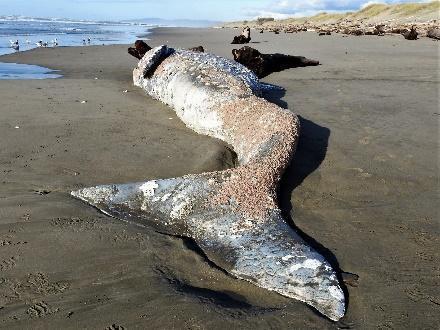 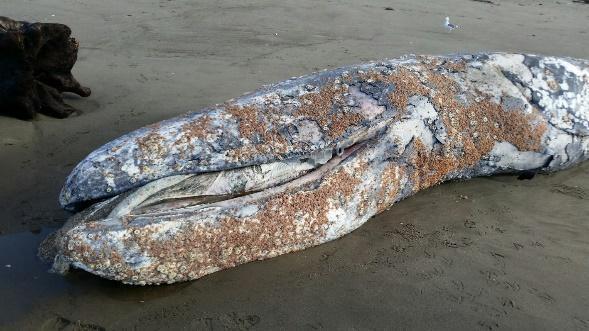 | | | |

**Other characteristics to consider/document-**

- Dorsal recumbency photos/criteria (**if you have heavy machinery - please take photos of both sides)
- Visible temporal mandibular joint? Concave/flat/convex mandible/throat area?
- Visible neck? (have to be careful with floaters because skin can expand, larynx full of other material)
- Neck width (from aerial photos?)
- Prominent post-anal cyst? (does this change with age?)
- Bony pectoral/scapular area

**Table A2 - Example Nutritional Body Condition Status Score (Findings highlighted in yellow)**

| **No.** | **External/Internal** | **Feature** | **1 Emaciated (Poor)** | **2 Thin (Fair)** | **3 Average (Moderate)** | **4 Fat (Good/Excellent)** | **CBD** |
| --- | --- | --- | --- | --- | --- | --- | --- |
| 1 | External | Nuchal fat pad area | Marked dipping | Slight dip | Flat | Convex |  |
| 2 | External | Vertebral processes | Prominent processes | Shape detectable | Not visible | Very rounded profile |  |
| 3 | External | Epaxial muscle profile (thoracic and lumbar views) | Sharply concave musculature | Slight dipping of the epaxial areas | Flat | Rounded convex (add new photo) |  |
| 4 | External | Scapulae prominence **(Live whales only)** | Very visible | Just visible | Not visible | Not visible (see nuchal fat photo above) | CBD |
| 5 | Internal | Blubber Characteristics - *Blubber Oiliness* | Watery or Dry | Low oil | Moderately oil (can see/feel oil but not dripping) | Very oily, oil drips when cut |  |
|  | Internal | *Blubber Texture* | Very fibrous | Moderately fibrous | Somewhat to moderately pliable | Very pliable |  |
| 6 | Internal | Subcutaneous fat | None | Scant | Moderate | Abundant |  |
| 7 | Internal | Fat in coronary groove, around kidneys, in mesentery, omental and mediastinum | None | Scant | Moderate | Abundant |  |
|  | **Other Features** | **Y/N/CBD** |  |  |  |  |  |
|  | Serous Atrophy of Fat | N |  |  |  |  |  |
|  | Blubber Color? | Y, Salmon color |  |  |  |  |  |
|  | Blubber Red-Band? | Y, Outer-layer band |  |  |  |  |  |
|  | Cyamid load? | Y, Moderate load, head |  |  |  |  |  |
|  | Gastrointestinal Contents? | None |  |  |  |  |  |
|  | Gastrointestinal Parasites? | None |  |  |  |  |  |
|  | Lipid Data? | NA |  |  |  |  |  |
|  | Histologic Nutritional Information | NA |  |  |  |  |  |
|  | Overall Body Shape-Snake-like | N |  |  |  |  |  |
|  | **Overall Score** |  |  | **2-Thin (Fair)** |  |  |  |
