# Supplementary material for: Gray whale (Eschrichtius robustus) post-mortem findings from December 2018 through 2021 during the Unusual Mortality Event in the Eastern North Pacific
Source: PLoS One. 2024 Mar 27;19(3):e0295861. doi: 10.1371/journal.pone.0295861 (PMC10971505; doi:10.1371/journal.pone.0295861)
Supplement: S2 Appendix — Case Definition: Killer Whale Predation as the Cause of Death in Gray Whales in Alaska. (DOCX) [file pone.0295861.s002.docx]


**S2. Case Definition:**

Killer Whale Predation as the Cause of Death in Gray Whales in Alaska

Kate Savage, Mandy Keogh, Mandy Migura, Jamie Musbach, Denise Greig, Lance Barrett-Lennard Kerri Danil, Padraig Duignan, Moe Flannery, Jon Ford, Barbie Halaska, Jessie Huggins, Craig Matkin, Stephen Raverty, Jim Rice, Gay Sheffield, Raphaela Stimmelmayr, Amy Willoughby, Deborah Fauquier.

1. **Background**

Since January of 2019, increased mortalities of eastern North Pacific gray whale (*Eschrichtius robustus*) have been documented along the west coast of North America, from Mexico to Alaska. In May 2019, NOAA Fisheries declared the rise in the number of strandings (i.e., mortalities) an Unusual Mortality Event (UME), which has persisted into 2022. A previous gray whale UME was declared along the West Coast from Mexico to Alaska during 1999-2000, but the cause was not determined (Gulland et al. 2001).

Most gray whales migrate almost 20,000 km round-trip between their wintering grounds in the warm waters off the Baja California peninsula Mexico, through the coastal waters of California, Oregon, Washington, and British Columbia, to their feeding grounds in high-latitude cooler waters spanning from the northern regions of British Columbia to Arctic Alaska. These areas also fall within the range of transient or Bigg’s killer whales (*Orcinus orca*), known predators of large cetaceans, including gray whales. The overlap of ranges is extensive and predation events have been documented throughout the migratory corridor, wintering grounds, and feeding areas (Lowry et al. 1987; Melnikov & Zagrebin 2005; Matkin et al. 2007; Ford & Reeves 2008; Barrett-Lennard et al. 2011; Matkin & Durban 2013; Willoughby et al. 2022).

A central goal of the UME investigative process includes the evaluation of potential causes of mortality. In 2021, members of the gray whale UME investigative team formed a subgroup to discuss signalment, geographic location, and which gross lesions may constitute evidence of killer whale predation as a cause of death (COD). The subgroup, comprised of biologists, veterinarians, and veterinary pathologists, formulated a case definition for describing the gross lesions and inferring a weight of evidence of predation events. Consistent evaluations of the lesions observed on gray whale carcasses would help interpret whether killer whale predation as the COD was confirmed, probable, suspected, improbable, or could not be determined (CBD).

Numerous organizations respond to gray whale stranding events, evaluate body condition, and document and assess signs of trauma and predation. Observations may be based on imagery, and when available, necropsy findings. In remote locations, documentation and evaluation of lesions and presumed COD for whale carcasses may rely solely on imagery; thus, the Case Definition must also apply to instances when only aerial or on-site photographic documentation is available.

It is important to note that lesions associated with killer whale predation are not specific to gray whales. The same characteristic lesions are observed on other large cetaceans such as fin, humpback, bowhead, minke, and sperm whales, and the Case Definition may eventually be applied to other large whale species.

1. **Case Definition**

A case definition of killer whale predation as a cause of death in gray whales includes the following:

1. **Signalment**

Lesions suggestive of killer whale predation have been documented in all age classes and sexes of gray whales. Calves and juveniles are often singled out for attack.

1. **Epidemiology/Geographic Location**

Killer whale/gray whale predation events are more commonly observed at some locations at certain times along the gray whale migratory route, e.g., Monterey Bay, California and Unimak Pass, Alaska.

1. **Clinical Signs**

Due to the rare and opportunistic nature of observing a predation event, clinical signs during or after attack have not been well or consistently documented. Whales being attacked may leave a trail of blood or exhibit signs of distress, increased ventilation efforts and rates, lethargy, obtund (dull, reduced level of awareness), or evasive swimming into shallow waters (Ford & Reeves 2008; Barrett-Lennard et al. 2011; Matkin & Durbin 2013). Mothers are highly protective and aggressively defensive, and may try to position themselves between calves or juveniles and predators (Ford & Reeves 2008; Barrett-Lennard et al. 2011; Matkin & Durbin 2013).

1. **External Examination/Gross Necropsy Findings**

Knowledge of killer whale attack strategies help to understand and validate external and internal injuries/lesions on gray whales associated with predation events. A multitude of possible predator/prey scenarios require consideration.

Documented predation events on large whales indicate that killer whales employ different tactics (herding, ramming, immobilizing, submerging) to subdue large whales and subsequently kill their prey (e.g., Melnikov & Zagrebin 2005). Killer whales often separate a targeted whale from its group, or a calf from its mother. Killer whales may strategically herd their target for presumably better access. Killer whales will forcefully ram their prey laterally or ventrally, which can cause bone fractures, contusions, or internal hemorrhaging from lacerated internal organs. They may attempt to immobilize larger whales by biting and holding on to the whale’s flukes, pectoral fins, and/or the tip of the rostrum, and then attempting to drown their prey by leaping onto its dorsum to submerge the whale before it can take a breath or to attempt to block its blowhole. Killer whales may make contact with their prey and bite chunks of tissue, leaving bite wounds or peeling off layers of skin (“skin” hereafter referring to the top two layers, i.e. epidermis and dermis, with the third layer, i.e. blubber or hypodermis, considered separately) which can result in evisceration or exteriorization of abdominal contents. They have been documented swimming off with large (1-2 m) pieces of tissue, sometimes with the pectoral fin attached. The tongue of their prey appears to be particularly preferred by killer whales. During the process of accessing and consuming the tongue, the jaw(s) of their prey may sustain damage such as soft tissue defects, fractured mandibles, separation of the mandibular symphysis, and/or partial or complete avulsion of the mandibles. Consequently, gray whale carcasses may have antemortem chunks of soft tissue missing, most commonly from the throat, tongue, and ventrum, including the peduncle. Strips of skin and blubber may be detached and lost or attached to the carcass by a narrow or broad pedicle.

Major lesions evident on stranded gray whale carcasses as a result of killer whale predation may include one or more of the following lesions:

- Missing portions of the tongue or the upper or lower jaw
- Semilunar bite wounds with variable amounts of tissue loss
- Detached and/or reflected sheets of skin and blubber
- Tooth impressions (“rake marks”)
- Fractured or avulsed bones, subcutaneous and muscular contusions, frank hemorrhage within the thoracic or abdominal cavities

Because the lesions listed above may vary with predation events, each was evaluated for:

- - 1. Mortality, i.e. is the lesion likely to result in death?
    2. Observability, i.e. is the lesion externally visible?
    3. Specificity, i.e. is the lesion specific to killer whale predation and, if not, what are the differentials?

Not all killer whale/gray whale interactions are fatal. Rake mark scars, evident as laminated white to gray-black curvilinear furrows most often on flukes in gray whales but also on the pectoral fins or throughout the torso, are present on many large whales, indicative of previous attempted predation and/or harassment encounters with killer whales (Corsi et al. 2021). Only three findings, missing tongue/jaw, bite wounds, and detached skin/blubber, are considered primary lesions because they are severe enough to cause mortality and are also externally observable. The remaining lesions are considered secondary or ancillary findings. Rake marks are considered to be secondary because they are unlikely to result in mortality. While fractured bones and contusions may be severe enough to cause death, they are most likely to be observed internally during a necropsy. Exceptions include the externally observable fractured mandibular or rostral bones associated with a missing tongue or jaw.

Some of these lesions may also result from traumatic events other than killer whale predation (e.g., vessel strike, attacks by other large marine predators). Therefore, before a level of certainty could be assigned, each major lesion was evaluated for specificity.

- - *Missing tongue or jaw (primary lesion)*

This lesion was determined to be pathognomonic, or highly specific, to killer whale predation events. Vessel strike and advanced decomposition were considered differentials. The subcommittee uniformly agreed that a missing tongue/jaw is not a feature of vessel strike. With respect to advanced decomposition, soft tissues can pull away from bone and the tongue may inflate/rupture as decomposition advances, possibly mimicking a missing tongue or jaw injury. With bloat, abdominal and thoracic viscera have been ejected from the oral cavity and may also result in artifactual loss of the tongue.

- - *Semilunar bite wounds with variable amounts of tissue missing (tissue defects) (primary lesion)*

This lesion type is not considered specific to killer whale predation. Attacks by other large marine predators, such as sharks, or post-mortem scavenging were considered possible differentials for bite wounds or missing tissue. However, in conjunction with unhealed rake marks, historical evidence of transient killer whales in the vicinity, or if the wounds match killer whale bite characteristics, the level of certainty of killer whale predation increases. With sharp, serrated and pointed teeth, evidence of shark predation or scavenging includes a scooped wound with clean and incised edges. Killer whale teeth are duller and more conical, leaving rake marks or wounds with ragged, torn and more irregular edges.

- - *Peeled skin including blubber (primary lesion)*

This lesion type is not considered specific to killer whale predation. It should be noted that the three skin layers, epidermis, dermis and hypodermis (i.e. blubber) is peeled as a unit and not to be confused with epidermal peeling or sloughing associated with decomposition. Ship or propeller strike, attacks by other large marine predators, and advanced decomposition were potential differentials for detached blubber and skin. However, in conjunction with fresh rake marks, the certainty of killer whale predation increases.

- - *Killer Whale Rake marks (secondary/ancillary lesion)*

Rake marks are defined as parallel superficial linear lacerations, approximately 2-4 cm apart, matching the tooth pattern of killer whales. Rake marks are most often found on the trailing edge of the flukes, fluke tips and flippers; however, raking can occur anywhere on the gray whale’s body. Non-healed (acute) rake marks are those where some level of laceration is still evident and non-pigmented (white) scar tissue has not yet formed. The freshness of rake marks may also be documented through histopathology where hemorrhage and edema may be evident in subjacent tissue. Rake marks are considered important ancillary findings because they are relatively common in killer whale predation events, and predation skill-building exercises for young killer whales, but are not known to cause sufficient injury resulting in mortality. The most likely differential might be contingent on brown and polar bear claw marks from scavenging but these would likely be found on different locations on the carcass (i.e., less likely to occur on flukes and pectoral fins) and there would not be antemortem soft tissue reaction on gross or histological examination. The presence of bear paw prints and/or feces around the carcass would offer further evidence of bear scavenging. Ante- or postmortem shark attack may be differentiated by more triangular than conical tooth profiles along the wound margins and the location of postmortem scavenging dependent upon carcass orientation in the water, e.g. lateral or dorsal if the carcass is floating belly-up. If fresh rake marks are the only lesions found on a carcass with no other indication of killer whale presence, they may be considered an incidental finding

- - *Contusions or fractured bones (secondary/ancillary lesion)*

Contusions or fractured bones are the least specific index of killer whale predation and commonly occur from other types of blunt trauma such as a vessel strike. Contusions or fractures, in conjunction with other primary or secondary lesions associated with killer whale predation would increase the level of certainty. Fractured bones and rupture of the mandibular symphysis from predation events must be differentiated from vessel strikes and other forms of blunt force trauma, particularly in active shipping lanes.

1. **Diagnostic Testing (if any)**
2. **Histological Findings**

Histological findings may include antemortem evidence of a vital response, such as fluid accumulation (edema), hemorrhage or acute inflammation acute cellular infiltrates (suppurative) associated with rake marks, semilunar bite wounds, or other traumatized tissue. In some cases, imbibed water from disruption of the epidermis, microbial colonization, and post mortem decomposition may impede microscopic assessment of the tissues.

1. **Environmental Data or Testing (if any)**

Presence of killer whales at or near the mortality site within the general or suspected time frame of death.

1. **Confirmed vs Probable vs Suspect vs Improbable vs CBD Killer Whale Predation Cases**

If a solitary primary or concurrent primary and secondary lesions substantiate killer whale attacks, then the likelihood of killer whale predation as the COD was assigned as confirmed, probable, suspected, improbable or could not be determined. However, several considerations influence the evaluation of post mortem and photographic findings and assignment of certainty.

- To assign killer whale predation as a COD, the severity of the primary lesions must be lethal.
- Changes associated with advanced decomposition may account for two of the primary lesions and hamper post mortem evaluation of the carcass. Valid lesions must be discernable from advanced decomposition and/or scavenging. For example, other than the area of the lesion, the structural integrity of the integument and skeleton must be adequately preserved.
- Necropsies, or at least a hands-on examination by experienced prosectors, should be performed whenever possible.
- In lieu of a hands-on examination, imagery may be used for evaluation. However, imagery must be of sufficient quality and views of the carcass are important. Factors to consider include focus, resolution, carcass not buried or floating too deep in the water etc.
  - The level of certainty regarding the classification of killer whale predation as COD may increase if additional data are available, such as the documented presence of killer whales in the area or bite wound characteristics matching those of killer whales.
- A pursued whale may strand alive, either in an attempt to evade the attack and/or due to injuries associated with a predation event. In these cases, rake marks and/or bleeding and fractured bones may be the only indications of attempted or confirmed killer predation.

Based on the considerations listed above, lesions observed either during a necropsy or in imagery can be assigned one of five levels of certainty relative to the likelihood of killer whale predation.

1. ***Confirmed cases***
2. Killer whales are observed attacking a live gray whale and the carcass is positively identified.
3. ***Probable cases***
4. A dead gray whale is observed with missing tongue and/or jaw(s); or
5. A dead gray whale is observed with at least two of the following lesions: semilunar bite wounds/chunks of fresh tissue missing; peeled or stripped blubber and skin; non-healed rake marks; and/or contusions/fractured bones.
6. ***Suspected cases***
7. A dead gray whale is observed with semilunar bite wounds/chunks of fresh tissue missing; or
8. A dead gray whale is observed with only peeled blubber and skin; or
9. A dead gray whale is observed with likely primary lesions, but quality of imagery, view of carcass or other factors prohibit certainty.
10. ***Improbable cases***
11. None of the criteria for confirmed, probable, suspected, or could not be determined are met.

1. ***Could not be determined***
2. Advanced decomposition, scavenging, and/or low-quality imagery do not impair an accurate evaluation; however, other factors (e.g., differentials) preclude assessment or lead to significant uncertainty.
3. Advanced decomposition, scavenging, and/or low-quality imagery preclude accurate evaluation.

**References:**

Baird, R.W., McSweeney, D.J., Bane, C., Barlow, J., Salden, D.R., Antoine, L.K., LeDuc, R.G. and D.L. Webster. 2006. Killer whales in Hawaiian waters: information on population identity and feeding habits. *Pacific Science* 60(4): 523-530.

Barrett-Lennard, L.G., Matkin, C.O., Durban, J.W., Saulitis, E.L., and D. Ellifrit. 2011. Predation on gray whales and prolonged feeding on submerged carcasses by transient killer whales at Unimak Island, Alaska. *Mar. Ecol. Prog. Ser*. 421, 229–241. doi:10.3354/meps08906

Corsi, E., J. Calambokidis, K.R. Flynn, & G.H. Steiger. 2021. Killer Whale Predatory Scarring on Mysticetes: A Comparison of Rake Marks Among Blue, Humpback, and Gray Whales in the Eastern North Pacific. Marine Mammal Science 38(1), 223-234. doi: 10.1111/mms.12863

Ferguson, S.H., Higdon, J.W., and K.H. Westdal. 2012. Prey items and predation behavior of killer whales (*Orcinus orca*) in Nunavut, Canada based on Inuit hunter interviews. *Aquat. Biosyst.* 8, 1–16. doi:10.1186/2046-9063-8-3

Ford, J.K.B., Ellis, G.M., Matkin, D.R., Balcomb, K.C., Briggs, D. and A.B. Morton. 2005. Killer whale attacks on minke whales: prey capture and antipredator tactics. *Marine Mammal Science* 21(4): 603-618.

Ford, J.K.B. and R.R. Reeves. 2008. Fight or flight: antipredator strategies of baleen whales. *Mammal Rev.* 38(1), 50-86.

George, J.C., Sheffield, G., Reed, D.J., Tudor, B., Stimmelmayr, R., Person, B.T., Sformo, T., and R. Suydam. 2017. Frequency of injuries from line entanglements, killer whales, and ship strikes on Bering-Chukchi-Beaufort Seas bowhead whales. *Arctic* 70, 37–46. doi:10.14430/arctic4631

Gulland, F.M.D., Perez-Cortes, M., Urban, R., Rojas-Bracho, L., Ylitalo, G., Norman, S.S., Muto, M.M., Rugh, D.J., Kreuder, C., and T. Rowles. 2001. Eastern North Pacific Gray Whale (*Eschrichtius robustus*) Unusual Mortality Event, 1999-2000. U.S. Dep. Commer., NOAA Technical Memorandum NMFS-AFSC-150, 34 pp

Lowry, L.F., Nelson, R.R., and K.J. Frost. 1987. Observations of killer whales, *Orcinus orca*, in Western Alaska: Sightings, strandings, and predation on other marine mammals. *The Canadian Field-Naturalist* 101:6-12.

Matkin, C.O., Barrett-Lennard, L., Yurk, H., Ellifrit, D., and A.W. Trites. 2007. Ecotypic variation and predatory behavior among killer whales (*Orcinus orca*) off the eastern Aleutian Islands, Alaska. *Fish. Bull.* 105, 74-87.

Matkin, C. and J. Durban. 2013. Gray Whale Killers. In “Whalewatcher Gray Whale From Devilfish to Gentle Giants”, *Journal of the American Cetacean Society* 42(1), 72 pp.

Mehta, A.V., Allen, J.M., Constantine, R., Garrigue, C., Jann, B., Jenner, C., Marx, M.K., Matkin, C.O., Mattila, D.K., Minton, G., Mizroch, S.A., Olavarria, C., Robbins, J., Russell, K.G., Seton, R.E., Steiger, G.H., Vikingsson, G.A., Wade, P.R., Witteveen, B.H., and P.J. Clapham. 2007. Baleen whales are not important as prey for killer whales *Orcinus orca* in high latitude regions. *Marine Ecology Progress Series* 348, 297-307.

Melnikov, V.V. and I.A. Zagrebin. 2005. Killer whale predation in coastal waters of the Chukotka Peninsula. *Marine Mammal Science* 21(3), 550-556.

NOAA Fisheries. Killer Whale https://www.fisheries.noaa.gov/species/killer-whale [accessed August 8, 2021]

Pitman, R.L., Totterdell, J.A., Fearnbach, H., Ballance, L.T., Durban, J.W., Kemps, H. 2015. Whale killers: Prevalence and ecological implications of killer whale predation on humpback whale calves off Western Australia. *Mar. Mammal Sci.* 31, 629–657. doi:10.1111/mms.12182

Reinhart, N.R., Ferguson, S.H., Koski, W.R., Higdon, J.W., LeBlanc, B., Tervo, O., Jepson, P.D. 2013. Occurrence of killer whale *Orcinus orca* rake marks on Eastern Canada West Greenland bowhead whales *Balaena mysticetus*. *Polar Biol.* 36, 1133–1146. doi:10.1007/s00300-013-1335-3

Visser, I.N., Zaeschmar, J., Halliday, J., Abraham, A., Ball, P., Bradley, R., Daly, S., Hatwell, T., Johnson, T., Johnson, W., Kay, L., Maessen, T., McKay, V., Peters, T., Turner, N., Umuroa, B., Pace, D.S., 2010. First record of predation on false killer whales (*Pseudorca crassidens*) by killer whales (*Orcinus orca*). *Aquat. Mamm.* 36, 195–204. doi:10.1578/AM.36.2.2010.195

Weller, D.W., Bradford, A.L., Lang, A.R., Burdin, A.M., and Brownwell, R.L. 2018. Prevalence of killer whale tooth rake marks on gray whales off Sakhalin Island, Russia. *Aquatic Mammals* 44(6):643–652. https://doi.org/10.1578/AM.44.6.2018.643

Willoughby, A.L., Ferguson, M.C., Stimmelmayr, R., Clarke, J.T., Brower, A.A., 2020. Bowhead whale (*Balaena mysticetus*) and killer whale (*Orcinus orca*) co-occurrence in the U.S. Pacific Arctic, 2009–2018: evidence from bowhead whale carcasses. *Polar Biol*. 43, 1669–1679. doi:10.1007/s00300-020-02734-y

Willoughby, A.L., Stimmelmayr, R., Brower, A.A. Clarke, J.T, Ferguson, M.C. 2022. Gray whale (*Eschrichtius robustus*) and killer whale (*Orcinus orca*) co-occurrence in the eastern Chukchi Sea, 2009–2019: evidence from gray whale carcasses observed during aerial surveys. *Polar Biol* 45:737–748 (2022). https://doi.org/10.1007/s00300-022-03015-6

Young, B.G., Fortune, S.M.E., Koski, W.R., Raverty, S.A., Kilabuk, R., Ferguson, S.H., 2020. Evidence of killer whale predation on a yearling bowhead whale in Cumberland Sound, Nunavut. *Arct. Sci.* 6, 53–61. doi:10.1139/as-2019-0014
