# Supplementary material for: Gray whale (Eschrichtius robustus) post-mortem findings from December 2018 through 2021 during the Unusual Mortality Event in the Eastern North Pacific
Source: PLoS One. 2024 Mar 27;19(3):e0295861. doi: 10.1371/journal.pone.0295861 (PMC10971505; doi:10.1371/journal.pone.0295861)
Supplement: Attachment — Submitted filename: POLS ONE-D-23-22593_reviewer - Comments.pdf [file pone.0295861.s003.pdf]

PLOS ONE

Gray whale (Eschrichtius robustus) post-mortem findings from 2018-2021 during the Unusual Mortality Event in the Eastern North Pacific

--Manuscript Draft--

**Manuscript Number:**	PONE-D-23-22593
**Article Type:**	Research Article
**Full Title:**	Gray whale (Eschrichtius robustus) post-mortem findings from 2018-2021 during the Unusual Mortality Event in the Eastern North Pacific
**Short Title:**	Post mortem findings of a Gray whale unusual mortality event in the Eastern North Pacific
**Corresponding Author:**	Stephen Raverty<br>Animal Health Centre<br>Abbotsford, British Columbia CANADA
**Keywords:**	Stranding, Health, Nutrition, Eastern North Pacific Gray Whales, Conservation
**Abstract:**	Beginning in December 2018, increased numbers of gray whale (Eschrichtius robustus) strandings were reported along the west coast of Mexico, the United States, and Canada, prompting declaration of a gray whale Unusual Mortality Event (UME) by the United States National Marine Fisheries Service. Although stranding numbers declined in 2020 and 2021 from a peak in 2019, the UME is still ongoing as of spring 2023. Between 17 December 2018 and 31 December 2021, 503 animals stranded along the west coast of North America, with 226 strandings in Mexico, 71 in California, 12 in Oregon, 56 in Washington, 21 in British Columbia, and 117 in Alaska. These strandings included 187 males, 167 females, and 149 whales of undetermined sex; and 193 adults, 194 subadults, 40 calves, 1 fetus, and 75 whales of undetermined age class. We report on 61 of the 503 carcasses (12%) that had external and internal gross necropsy and/or histopathology data: of these 61 whales, findings that contributed to death were identified in 33 (54%). Sixteen of the 61 (26%) were severely emaciated. Gross lesions of blunt force trauma consistent with vessel strike were identified in 11 of the 61 animals (18%), only two of which were emaciated. Two whales (3%) had evidence of active entanglements and one whale died from entrapment. Signs of killer whale (Orcinus orca) interaction were documented in 19 of the 61 animals; five were judged to be from recent interactions and three (5%) could be determined as likely to have contributed to mortality. A specific cause of death could not be identified in 28 of 61 whales (46%). Additionally, logistical challenges and the advanced state of decomposition of most examined carcasses precluded detection of potential infectious or toxic causes of morbidity or mortality. Up to 2016, the eastern North Pacific population of gray whales had generally been increasing since the cessation of historic whaling and a prior UME in 1999-2000. However, recent abundance and calf production estimates have declined, a trend that overlaps the current UME. The relative contributions of carrying capacity, environmental change, prey shifts, and infectious, toxic, and other processes to the increased gray whale mortalities have not yet been resolved. Nevertheless, the marked temporal increase in strandings for the 1999-2000 and current UME, including findings of malnutrition in some of the whales, along with low calf production, likely represents consequences of complex and dynamic ecological interactions in the ocean impacting the eastern North Pacific gray whale population.
**Order of Authors:**	Stephen Raverty
	Padraig Duignan
	Denise Greig
	Jessica Huggins
	Kathy Burek-Huntington
	Michael Garner
	John Calambokidis

Powered by Editorial Manager® and ProduXion Manager® from Aries Systems Corporation

	Paul Cottrell
	Kerri Danil
	Dalin D'Alessandro
	Deborah Duffield
	Moe Flannery
	Frances Gulland
	Barbie Halaska
	Dyanna Lambourn
	Taylor Lehnhart
	Jorge Urban Ramirez
	Teri Rowles
	James Rice
	Kate Savage
	Kristin Wilkinson
	Justin Greenman
	Justin Viezbicke
	Bredan Cottrell
	P Dawn Goley
	Maggie Martinez
	Deborah Fauquier
**Additional Information:**	
**Question**	**Response**
**Financial Disclosure**<br><br>Enter a financial disclosure statement that describes the sources of funding for the work included in this submission. Review the submission guidelines for detailed requirements. View published research articles from *PLOS ONE* for specific examples.<br><br>This statement is required for submission and **will appear in the published article** if the submission is accepted. Please make sure it is accurate.	This study was funded in part by the Unusual Mortality Working Group, the National Marine Mammal Response Program, Prescott Funds and the Department of Fisheries and Oceans

**Unfunded studies**<br>Enter: *The author(s) received no specific funding for this work.*<br><br>**Funded studies**<br>Enter a statement with the following details:<br>• Initials of the authors who received each award<br>• Grant numbers awarded to each author<br>• The full name of each funder<br>• URL of each funder website<br>• Did the sponsors or funders play any role in the study design, data collection and analysis, decision to publish, or preparation of the manuscript?<br>• **NO** - Include this sentence at the end of your statement: *The funders had no role in study design, data collection and analysis, decision to publish, or preparation of the manuscript.*<br>• **YES** - Specify the role(s) played.<br><br>* typeset	
**Competing Interests**<br><br>Use the instructions below to enter a competing interest statement for this submission. On behalf of all authors, disclose any competing interests that could be perceived to bias this work—acknowledging all financial support and any other relevant financial or non-financial competing interests.<br><br>This statement is required for submission and **will appear in the published article** if the submission is accepted. Please make sure it is accurate and that any funding sources listed in your Funding Information later in the submission form are also declared in your Financial Disclosure statement.<br><br>View published research articles from *PLOS ONE* for specific examples.	Dr Kathy Burek owns and operations the Alaska Veterinary Pathology Services, Eagle River, AK and Dr Michael Garner owes the Northwest ZooPath, Monroe, WA

Powered by Editorial Manager® and ProduXion Manager® from Aries Systems Corporation

**NO authors have competing interests**<br><br>Enter: *The authors have declared that no competing interests exist.*<br><br>**Authors with competing interests**<br><br>Enter competing interest details beginning with this statement:<br><br>*I have read the journal's policy and the authors of this manuscript have the following competing interests: [insert competing interests here]*<br><br>* typeset	
**Ethics Statement**<br><br>Enter an ethics statement for this submission. This statement is required if the study involved:<br><br>• Human participants<br>• Human specimens or tissue<br>• Vertebrate animals or cephalopods<br>• Vertebrate embryos or tissues<br>• Field research<br><br>Write "N/A" if the submission does not require an ethics statement.<br><br>General guidance is provided below. Consult the submission guidelines for detailed instructions. **Make sure that all information entered here is included in the Methods section of the manuscript.**	This study focused on post mortem examination of dead marine mammals and is exempt from IACUC review.

**Format for specific study types**<br><br>**Human Subject Research (involving human participants and/or tissue)**<br>• Give the name of the institutional review board or ethics committee that approved the study<br>• Include the approval number and/or a statement indicating approval of this research<br>• Indicate the form of consent obtained (written/oral) or the reason that consent was not obtained (e.g. the data were analyzed anonymously)<br><br>**Animal Research (involving vertebrate animals, embryos or tissues)**<br>• Provide the name of the Institutional Animal Care and Use Committee (IACUC) or other relevant ethics board that reviewed the study protocol, and indicate whether they approved this research or granted a formal waiver of ethical approval<br>• Include an approval number if one was obtained<br>• If the study involved *non-human primates*, add *additional details* about animal welfare and steps taken to ameliorate suffering<br>• If anesthesia, euthanasia, or any kind of animal sacrifice is part of the study, include briefly which substances and/or methods were applied<br><br>**Field Research**<br><br>Include the following details if this study involves the collection of plant, animal, or other materials from a natural setting:<br>• Field permit number<br>• Name of the institution or relevant body that granted permission	
**Data Availability**<br><br>Authors are required to make all data underlying the findings described fully available, without restriction, and from the time of publication. PLOS allows rare exceptions to address legal and ethical concerns. See the PLOS Data Policy and FAQ for detailed information.	Yes - all data are fully available without restriction

A Data Availability Statement describing where the data can be found is required at submission. Your answers to this question constitute the Data Availability Statement and **will be published in the article**, if accepted.<br><br>**Important:** Stating 'data available on request from the author' is not sufficient. If your data are only available upon request, select 'No' for the first question and explain your exceptional situation in the text box.<br><br>Do the authors confirm that all data underlying the findings described in their manuscript are fully available without restriction?	
**Describe where the data may be found in full sentences. If you are copying our sample text, replace any instances of XXX with the appropriate details.**<br><br>• If the data are **held or will be held in a public repository**, include URLs, accession numbers or DOIs. If this information will only be available after acceptance, indicate this by ticking the box below. For example: *All XXX files are available from the XXX database (accession number(s) XXX, XXX.).*<br>• If the data are all contained **within the manuscript and/or Supporting Information files**, enter the following: *All relevant data are within the manuscript and its Supporting Information files.*<br>• If neither of these applies but you are able to provide **details of access elsewhere**, with or without limitations, please do so. For example:<br><br>*Data cannot be shared publicly because of [XXX]. Data are available from the XXX Institutional Data Access / Ethics Committee (contact via XXX) for researchers who meet the criteria for access to confidential data.*<br><br>*The data underlying the results presented in the study are available from (include the name of the third party*	https://www.fisheries.noaa.gov/national/marine-life-distress/2019-2023-gray-whale-unusual-mortality-event-along-west-coast-and<br>Additional data is available in the Supplements Sections

Powered by Editorial Manager® and ProduXion Manager® from Aries Systems Corporation

and contact information or URL).<br>• This text is appropriate if the data are owned by a third party and authors do not have permission to share the data.<br><br>* typeset	
Additional data availability information:	Tick here if your circumstances are not covered by the questions above and you need the journal’s help to make your data available.

Manuscript

Click here to access/download;Manuscript;Gray whale UME pathology paper July 16, 2023.docx

Gray whale (*Eschrichtius robustus*) post-mortem findings from 2018-2021 during the Unusual Mortality Event in the Eastern North Pacific

Stephen Raverty[1¶*#a], Pádraig Duignan[2¶], Denise Greig[3,10¶], Jessica L Huggins[4], Kathy Burek Huntington[5], Michael Garner[6], John Calambokidis[4], Paul Cottrell[7], Kerri Danil[8], Dalin D'Alessandro[9], Deborah Duffield[9], Moe Flannery[10], Frances MD Gulland[11], Barbie Halaska[2], Dyanna M Lambourn[12], Taylor Lehnhart[7], Jorge Urbán R.[13], Teri Rowles[3], James Rice[14], Kate Savage[15], Kristin Wilkinson[16], Justin Greenman[17], Justin Viezbicke[17], Brendan Cottrell[18], P. Dawn Goley[19], Maggie Martinez[2], and Deborah Fauquier[3]

[1]Animal Health Center, Abbotsford, BC, Canada
[2]The Marine Mammal Center, Sausalito, CA, United States of America
[3]Ocean Associates, Inc. under contract to Office of Protected Resources, National Marine Fisheries Service, National Oceanic and Atmospheric Administration, Silver Spring, MD, United States of America
[4]Cascadia Research Collective, Olympia, WA, United States of America
[5]Alaska Veterinary Pathology Services, Eagle River, AK, United States of America
[6]Northwest ZooPath, Monroe, WA, United States of America
[7]Fisheries and Oceans Canada, Vancouver, BC, Canada
[8] Southwest Fisheries Science Center, National Marine Fisheries Service, National Oceanic and Atmospheric Administration, La Jolla, CA, United States of America
[9]Portland State University, Department of Biology, Portland, OR, United States of America
[10]California Academy of Sciences, San Francisco, CA, United States of America
[11]Wildlife Health Center, School of Veterinary Medicine, UC Davis, CA, United States of America
[12]Washington Department of Fish and Wildlife, Marine Mammal Investigations, Lakewood, WA, United States of America
[13]Departamento de Ciencias Marinas y Costeras, Universidad Autónoma de Baja California Sur, La Paz, Mexico
[14]Oregon State University Marine Mammal Institute, Hatfield Marine Science Center, Newport, OR, United States of America
[15]Alaska Biosystems under contract to Alaska Regional Office, National Marine Fisheries Service, National Oceanic and Atmospheric Administration, Juneau, AK, United States of America
[16]Protected Resources Division, West Coast Region, National Marine Fisheries Service, National Oceanic and Atmospheric Administration, Seattle, WA, United States of America
[17]Protected Resources Division, West Coast Region, National Marine Fisheries Service, National Oceanic and Atmospheric Administration, Long Beach, CA, United States of America
[18]Applied Remote Sensing Lab, Department of Geography, McGill University, Montreal, QC, Canada
[19]Cal Poly Humboldt, Department of Biological Sciences, Arcata, CA, United States of America

[#a] Current address: University of British Columbia, Vancouver, BC, Canada

***Correspondence:**
Stephen A Raverty
stephen.raverty@gov.bc.ca

Stephen Raverty, Pádraig Duignan, and Denise Greig contributed equally to the preparation of this manuscript.

Keywords (5-8): Strandings, Health, Nutrition, Eastern North Pacific Gray Whales, Conservation

Words in main body: 10245, Tables: 8, Figures: 7, Supplementary materials: 2 appendices

Abstract

Beginning in December 2018, increased numbers of gray whale (*Eschrichtius robustus*) strandings were reported along the west coast of Mexico, the United States, and Canada, prompting declaration of a gray whale Unusual Mortality Event (UME) by the United States National Marine Fisheries Service. Although stranding numbers declined in 2020 and 2021 from a peak in 2019, the UME is still ongoing as of spring 2023. Between 17 December 2018 and 31 December 2021, 503 animals stranded along the west coast of North America, with 226 strandings in Mexico, 71 in California, 12 in Oregon, 56 in Washington, 21 in British Columbia, and 117 in Alaska. These strandings included 187 males, 167 females, and 149 whales of undetermined sex; and 193 adults, 194 subadults, 40 calves, 1 fetus, and 75 whales of undetermined age class. We report on 61 of the 503 carcasses (12%) that had external and internal gross necropsy and/or histopathology data: of these 61 whales, findings that contributed to death were identified in 33 (54%). Sixteen of the 61 (26%) were severely emaciated. Gross lesions of blunt force trauma consistent with vessel strike were identified in 11 of the 61 animals (18%), only two of which were emaciated. Two whales (3%) had evidence of active entanglements and one whale died from entrapment. Signs of killer whale (*Orcinus orca*)

interaction were documented in 19 of the 61 animals; five were judged to be from recent interactions and three (5%) could be determined as likely to have contributed to mortality. A specific cause of death could not be identified in 28 of 61 whales (46%). Additionally, logistical challenges and the advanced state of decomposition of most examined carcasses precluded detection of potential infectious or toxic causes of morbidity or mortality. ~~Up to 2016, the eastern North Pacific population of gray whales had generally been increasing since the cessation of historic whaling and a prior UME in 1999-2000. However, recent abundance and calf production estimates have declined, a trend that overlaps the current UME. The relative contributions of carrying capacity, environmental change, prey shifts, and infectious, toxic, and other processes to the increased gray whale mortalities have not yet been resolved. Nevertheless, the marked temporal increase in strandings for the 1999-2000 and current UME, including findings of malnutrition in some of the whales, along with low calf production, likely represents consequences of complex and dynamic ecological interactions in the ocean impacting the eastern North Pacific gray whale pop~~ulation.

Introduction

There are two genetically distinct gray whale (*Eschrichtius robustus*) populations: the eastern north Pacific (ENP) and western north Pacific (WNP) [1]. The ENP whales range seasonally from wintering grounds off Mexico to primary feeding areas in Arctic waters, with the exception of a small subset, the Pacific Coast Feeding Group (PCFG). The PCFG whales are often observed feeding in summer and fall from northern California to southeastern Alaska [2], and the endangered WNP population have been observed feeding off Sakhalin Island, Russia [1]. Whales from both populations have been observed on the wintering grounds in Mexico and along the ENP migratory route, but the extent of mixing is not clear [1-4]. Heavily exploited

during the 1800s and 1900s by commercial whaling, the ENP population has made a strong recovery with abundance estimated in the mid-2010s at approximately 27,000 [6-7], the greatest number documented for this population [8]. Models of gray whale abundance and mortality estimated carrying capacity in recent years as close to 25,000 [9].

Gray whale abundance in the 1850s was thought to be less than 30,000 [10], although genetic studies have estimated higher numbers existed through evolutionary time [11-12]. The 2021/2022 ENP gray whale abundance estimate was 16,650, decreased from an estimated 20,580 whales in 2019/2020, and 26,960 whales in 2015/2016 [13]. Similar decreases in estimated abundance, from a time series since 1967, occurred between 1987/1988 and 1992/1993 and between 1997/1998 and 2000/2001, which coincided with the 1999-2001 UME [12].

Transformative ecosystem changes have occurred in the summer Bering Strait feeding grounds [14] with reported local declines in prey abundance and quality [15] which may have a direct impact on gray whale bioenergetics, fecundity, health, and homeostasis [16]. Variability in calf production in the ENP gray whale population over a 23-year period has been associated with the extent of seasonal ice cover in the Bering Sea in the previous feeding season and thought to reflect the greater spatial and temporal access to benthic feeding provided by lesser ice cover [17-18]. For the endangered western gray whale population, a correlation was observed between decreased calf production and a shorter foraging season (i.e. fewer ice free days) off Sakhalin Island [19]. Among the gray whales foraging on the PCFG feeding ground, decreased body condition was documented in years following poor upwelling conditions [16]. Periods of low calf production occurred during periods of increased mortality [20,13] and the most recent estimate of calf production for the Eastern North Pacific gray whale population was the lowest of the time series, with 217 calves in 2022 [21].

In addition to impacts of environmental change on calf production, increased vessel traffic and extensive commercial fisheries along migratory corridors throughout the western

seaboard of North America have been associated with vessel strikes and entanglements of gray whales [22-23]. These may be immediately lethal or incapacitate animals due to pain, secondary infection, and the incurred energetic costs of wound repair and resolution [24]. In a review of gray whale entanglements and vessel strikes in the north Pacific between 1924 and 2015, 40% of injuries and mortalities were due to net fisheries, 17% to pot fisheries, 22% were from unknown fisheries entanglements, and 19% were from vessel strike [23]. Other potential threats to gray whales include acute or chronic noise exposure, physical disturbances, disruption of feeding habitats, infectious diseases, harmful algal blooms, toxic spills, pollutants, predation, subsistence harvest, and scientific research [9].

From 1999 through 2000, an Unusual Mortality Event (UME) of gray whales occurred along the Pacific coast of North America with total strandings of 283 in 1999 and 368 in 2000 [25]. Only three whales were necropsied during this event and a cause of death for most whales was not documented but malnutrition was considered a prime contributor to the UME [25]. One animal had an encephalitis, another had heavy intestinal helminth infection, and a third had high levels of domoic acid, a biotoxin, in gut contents [26]. Historically, gray whales were thought to fast during their migration while resident on the wintering grounds [27]; however, more recent field observations indicate some animals actively forage during the southward and northward migrations [20, 28-30]. While the cause of the 1999-2000 mortality event was not determined, the combination of thin and emaciated stranded animals, field observations of migrating whales in poor body condition, reduced calf production, a population suspected to be nearing carrying capacity due to recent increases in abundance, and reduced prey densities in these years contributed to this dramatic event [25, 31-34].

Beginning in December 2018 and early 2019, increased numbers of gray whale strandings were reported in Mexico (MX) and the continental United States (US), including California (CA), Oregon (OR), and Washington (WA). As a result of the increase in strandings,

the United States National Marine Fisheries Service (NMFS) declared an UME in 2019 (https://www.fisheries.noaa.gov/national/marine-life-distress/2019-2023-gray-whale-unusual-mortality-event-along-west-coast-and). Designation of an UME resulted in a federal investigation that facilitated expert consultation, coordination of stranding and necropsy responses in three countries, and enhanced communication amongst stranding response teams. Media reports heightened public awareness for reporting of carcasses. This paper compiles and presents the gross and histologic findings from a subset of the gray whales that stranded and were examined along the North American west coast between December 2018 and December 2021.

Materials and Methods

Marine mammal mortalities observed by the public, biologists, research scientists, enforcement, Native American Tribes, First Nations, and indigenous communities were reported to Canadian, Mexican, or US regional marine mammal stranding response networks, and when feasible, trained personnel were mobilized to examine stranded whales. The teams typically included experienced field biologists, research scientists and veterinarians; basic data on these animals were collected [e.g., NMFS Level A data (NOAA Form 89-864; OMB Control No.0648-017)] including species, date, sex, age class, and stranding location and signs of human interaction (e.g., entanglement, vessel strike). Photographs of the carcasses from different angles were subsequently examined to assess the nutritional status, cyamid load, identifying features of individual animals, evidence of fisheries interactions, indication of killer whale attacks, and any other apparent external lesions.

Age classes were initially based on a combination of length from tip of the snout to the fluke notch (calf <7-8 m, yearling 8-9 m, subadult 9-11.1 m for males and 9-11.7 m for females,

adult ≥11.1 m for males and ≥ 11.7 m for females). These were subsequently modified depending on date and latitude of stranding, baleen plate length, and barnacle size, as calves and subadults in the lower latitudes were often shorter than the previously reported length ranges [27, 35-39]. Yearling and subadult age classes were combined because, outside Mexico, 1-2 year-olds could not be distinguished from 2-3 year olds. Age classes were finally categorized as calf/fetus (0-12 months), subadult (12 months-sexual maturity), and adult. The calf cohort includes fetuses from the calving lagoons in MX and a single fetus documented in British Columbia (BC), Canada in 2021 [40]. Carcasses were evaluated for nutritional status using a protocol (Appendix 1) established by a review of carcass photographs obtained at the time of necropsy, external assessment of the body condition, internal examination of epicardial and mesenteric fat stores, and microscopic evaluation of full thickness blubber sections for evidence of adipocyte atrophy or stromal collapse. As body position or posture, the stage of putrefaction, bloat, scavenging, and post mortem oil seepage from the blubber may alter the gross appearance and thickness of the blubber, microscopic findings of a disproportionate increase in stroma to adipocytes and fat atrophy were interpreted in context of the gross observations to assign the overall nutritional score for an animal. For the more challenging cases, gross and histopathology findings were reviewed by pathologists, in conjunction with field biologists and prosectors, to derive a nutritional score. Stranded animals were assigned into one of four nutritional scores: Emaciated (1), thin (2), average (3) and fat (4). Condition at initial observation was defined as alive, fresh dead, moderate decomposition, advanced decomposition, mummified, or undetermined [41].

Based on the carcass condition and access, post-mortem examinations (PMEs) were performed as thoroughly and systematically as possible. The extent of PME was contingent on multiple variables including, but not limited to, carcass access, state of decomposition, human safety, tides, time available due to daylight, weather and tides, available transport to floating animals, and whether the animal was beach-cast or floating offshore. For fresher floating

carcasses or those with gross evidence of traumatic injuries, efforts were made to recover and/or tow the animal ashore to a secure and accessible site. In some instances, examination of floating animals was limited to photographs above and under water using submerged waterproof cameras, and skin and blubber biopsy for genetics and histopathology. The cases discussed in this paper are those for which there was external and internal gross necropsy and/or histopathology data. Many other carcasses that we did not include were extensively sampled and/or were confirmed, probable, or suspect cases of human or killer whale interaction. As such, the numbers reported here are minimum numbers and represent a subset of the full UME response and investigation.

Depending on the orientation of the animal, typically the skin and blubber from the flanks was flensed, reflected, and the blubber was assessed for nutritional status and trauma. The abdominal and thoracic cavities were then incised, the ribs removed, body cavities examined, and appropriate tissue samples harvested. In some cases, access to internal organs was limited to "windows" or small incised portals that facilitated access to specific viscera [42]. Although response level varied, 61 animals that received external and some degree of internal examination were included here. Anthropogenic trauma from vessel strikes or entanglements was assessed and documented as described in [43] and is reported here for the 61 cases examined.

Killer whale predation as a cause of death was determined based on lesions that have been documented during observed predation events. Evidence of killer whale predation on whale carcasses includes missing portions of the tongue or the upper or lower jaw, semilunar bite wounds with variable amounts of tissue loss, detached and/or reflected sheets of blubber and skin, tooth impressions ("rake marks"), and fractured or avulsed bones, subcutaneous and muscular contusions, and frank hemorrhage within the thoracic or abdominal cavities and associated tissues [44-46]. Pre-mortem killer whale predation was differentiated from post-mortem scavenging by the presence of associated superficial or deep hemorrhage and edema in

the tissues and abrupt margins associated with the rake marks (lack of imbibed water or melanocyte degranulation).

After review of the literature reporting confirmed predation cases in Alaska (AK), a case definition was formulated to consistently evaluate the gross lesions, and assign a level of certainty associated with killer whale predation and mortality (Appendix 2). The levels were confirmed, probable, suspect, improbable, and could not be determined. We assigned these levels of certainty to cases with signs of recent killer whale interaction. Although the case definition can be used to review photos of dead floating and beach cast whales, here we evaluated killer whale interactions for the 61 cases for which we had external and internal gross or microscopic pathology data. The case definition has been tested using AK whales; it has not yet been broadly applied to other geographic areas.

We assessed pigment deposition along the wound margins, second intention healing, and varying degrees of re-epithelialization to discriminate a peracute injury, from a more chronic and resolving process that we considered incidental to mortality. In cases that presented in advanced autolysis, the wound margins were more closely scrutinized and if inconclusive, were not considered predation events. Also considered were circumstances associated with strandings (clinical history), field observations or reports of transient killer whales in the vicinity and near the time of the mortality, and exclusion of vessel strike and other disease processes.

If the animal was accessible and was fresh or in a moderate state of decomposition, formalin fixed and fresh tissue samples were collected, and forwarded to a diagnostic laboratory for analysis, or frozen and retained for follow up investigations. When feasible, samples including baleen, ear plug (wax), stomach contents, full thickness blubber, and ovaries were archived. Additional samples of select tissues and observed lesions from the fresher whales were fixed in 10 percent neutral buffered formalin for conventional histologic processing and microscopic evaluation by veterinary pathologists.

For this review of stranding and post-mortem records, the stranding location, demographic data (*e.g.*, NMFS Level A), and necropsy reports were uploaded to a shared Google drive and a spreadsheet of cases was maintained by responders. The gross and histologic findings were compiled by a research scientist and two veterinary pathologists with summary tables forwarded to regional stranding responders and investigative team members to check for accuracy. Reported stranding locations (in decimal degrees) were used to prepare maps in ArcGIS Pro 2.8.0.

Results

Stranding response and demographics

Between 17 December 2018 and 31 December 2021, 503 gray whale strandings were reported along the Pacific coast of MX (n=226 ~~or~~ 44.9% of total), the US (n=256, 50.9%) and Canada (n=21, 4.2%) (Table 1, Fig 1). Strandings occurred in each month of the year, with peak numbers in April (n=99 or 19.7%) (Table 2). The proportion of strandings by region, included 71 in CA (14.1% of strandings), 56 in WA (11.1%), 12 in OR (2.4%), 21 in BC (4.2%) and 117 in AK (23.3%) (Table 1). In contrast to BC, WA, OR, CA and MX, most of the reports for AK were from aerial surveys or opportunistic aerial sightings of floating or beach cast animals. Post-mortem exams from 21 of 71 (29.6%) animals that stranded in CA, 1 of 12 (8.3%) animals in OR, 26 of 56 (46.4%) animals in WA, 9 of 21 (42.8%) animals in BC, and 4 of 117 (3.4%) animals stranded in AK are reported here (Table 1). No PME data from the 225 animals stranded in MX are reported in this paper.

Table 1. Reported strandings (whales with findings reported in this paper) by location and year (17 December 2018 to 31 December 2021). MX = Mexico, CA=California, OR=Oregon, WA=Washington, BC=British Columbia, AK=Alaska.

Location	Number of Strandings per Year (Number of Post-mortem Exams)

	2019	2020	2021	Total	% Strandings
MX	83*	88	55	226	44.9%
CA	34 (12)	18 (2)	19 (7)	71 (21)	14.1%
OR	6 (1)	3	3	12 (1)	2.4%
WA	34 (16)	13 (8)	9 (2)	56 (26)	11.2%
BC	11 (3)	5 (4)	5 (2)	21 (9)	4.2%
AK	48 (4)	45	24	117 (4)	23.1%
**Total**	**216 (36)**	**172 (14)**	**115 (11)**	**503 (61)**	**100%**

*2 whales from December 2018 in Mexico are included in the 2019 tally

Table 2. Reported stranded gray whales (whales with findings reported in this paper) in Mexico, the United States and Canada by month. Animals are categorized by month and geographic location from south to north. MX = Mexico, CA=California, OR=Oregon, WA=Washington, BC=British Columbia, AK=Alaska.

Number of Strandings (Number of Post-mortem Exams)							
**Month**	MX	CA	OR	WA	BC	AK	**Total**
January	31	1	0	1	0	0	**33**
February	60	4	1	2 (1)	0	0	**67 (1)**
March	61	19 (4)	1	4 (3)	0	0	**85 (7)**
April	55	21 (10)	4	14 (7)	4 (3)	1	**99 (21)**
May	11	21 (4)	2	19 (10)	5 (1)	13 (2)	**71 (17)**
June	0	1 (1)	3 (1)	6 (2)	4 (1)	32 (1)	**46 (6)**
July	0	0	0	4 (2)	4 (3)	36 (1)	**44 (6)**
August	0	1	1	4 (1)	1	14	**21 (1)**
September	0	1	0	0	0	15	**16**
October	0	0	0	1	1 (1)	5	**7 (1)**
November	0	1 (1)	0	0	2	1	**4 (1)**
December	8	1 (1)	0	1	0	0	**10 (1)**

Fig. 1. Map of gray whale strandings from Dec 2018 through 2021. The map shows the locations of all 503 whales reported, and the vessel strike and emaciation cases among the 61 whales discussed in this paper. Inset maps show area details around Puget Sound and San Francisco Bay.

The demographics of the reported gray whales include 193 adults (38.4%), 194 subadults (38.6%), 41 (8.2%) calves (40) or fetuses (1), and 75 undetermined age (14.9%). The age class and number of animals varied by geographic region and reflected the stage of the migration cycle (Table 3). Among adults, there were 103 animals identified in MX, 31 in AK, 27 in CA, 22 in WA, 9 in BC and 1 in OR. In the subadult cohort, 98 strandings were recorded in MX, with 23 in AK, 27 in WA, 32 in CA, 9 in OR and 5 in BC. Calves were mostly encountered in MX (n=22) with fewer calves in AK (n=8), CA (n=6), WA (n=3) and OR (n=1); the only fetus was recorded in BC [37]. There were 187 males, 167 females and 149 whales of undetermined sex (Table 4).

Table 3. Reported stranded gray whales (whales with findings reported in this paper) in Mexico, the United States and Canada by age class and geographic location from south to north. MX = Mexico, CA=California, OR=Oregon, WA=Washington, BC=British Columbia, AK=Alaska.

Location	Age Class				
	Calf/Fetus	Subadult	Adult	Unknown	Totals
MX	22	98	103	3	226
CA	6 (2)	32 (8)	27 (11)	6	71 (21)
OR	1	9 (1)	1	1	12 (1)
WA	3	27 (10)	22 (16)	4	56 (26)
BC	1* (1)	5 (2)	9 (6)	6	21 (9)
AK	8	23 (2)	31 (2)	55	117 (4)
**Total**	**41 (3)**	**194 (22)**	**193 (35)**	**75**	**503 (61)**

*Indicates the one fetus, the other 40 whales were calves.

Table 4. Reported strandings (whales with findings reported in this paper) by sex and year. MX = Mexico, CA=California, OR=Oregon, WA=Washington, BC=British Columbia, AK=Alaska.

Location	Number of Strandings per Year (Number of Post-mortem Exams)			
	2019	2020	2021	Total
		Male		
MX	20	34	32	86
CA	10 (3)	7 (1)	10 (3)	27 (7)
OR	2	3	2	7
WA	14 (8)	5 (3)	3 (1)	22 (12)
BC	1 (1)	4 (3)	2 (2)	7 (6)
AK	9 (3)	20	10	39 (3)
		Female		
MX	52	25	16	92
CA	14 (9)	9 (1)	7 (4)	30 (14)
OR	3 (1)	0	0	3 (1)
WA	14 (8)	4 (4)	4 (1)	22 (13)
BC	6 (2)	0	1	7 (2)
AK	6 (1)	6	1	13 (1)
		Unknown		
MX	11	29	7	47
CA	10	2	2	14
OR	1	0	1	2
WA	6	4 (1)	2	12 (1)
BC	4	1 (1)	2	7 (1)
AK	33	19	13	65
**Total**	**216 (35)**	**172 (14)**	**115 (11)**	**503 (61)**

*2 whales from December 2018 in Mexico are included in the 2019 tally

Carcass decomposition varied by country and region and ranged from 10 mummified animals (2.0%), 251 (50.0%) in advanced decomposition, 175 (34.8%) in moderate decomposition, 51 (10.1%) fresh dead, and 3 (0.6%) that were initially observed alive (Table 5). Where the carcass code was scored as between decomposition states (e.g. 2.5 or 3.5, [38], the post-mortem state was assigned to the more advanced decomposition state for tabulation and analysis. No examinations were conducted on mummified carcasses. Post-mortem examinations were performed on 12 animals with advanced decomposition (code 4), 35 animals with moderate decomposition (code 3), and 14 fresh carcasses (code 2).

Table 5. Reported stranded gray whales (whales with findings reported in this paper) in Mexico, the United States and Canada by decomposition state and geographic location from south to north. MX = Mexico, CA=California, OR=Oregon, WA=Washington, BC=British Columbia, AK=Alaska. Decomposition state is recorded at the time of examination, however for whales that were not examined at all, it is the decomposition state at first observation.

Location	First observed alive	Fresh Dead	Moderate Decomposition	Advanced Decomposition	Mummified	Unknown	
MX	1	11	71	137	6		226
CA	1	16 (7)	38 (13)	16 (1)			71 (21)
OR		1	7	3 (1)		1	12 (1)
WA		3 (2)	27 (19)	24 (5)	2		56 (26)
BC	1	4 (3)	1 (2)	7 (4)		8	21(9)
AK		16 (2)	31 (1)	64 (1)	2	4	117 (4)
**Total**	**3**	**51 (14)**	**175 (35)**	**251 (12)**	**10**	**13**	**503 (61)**

Gross findings

Review of stranding datasheets, photographs, and reports of gross findings identified three dominant findings that likely contributed to death: emaciation, vessel strike, and signs of killer whale interaction (Table 6, Figs 1 and 2). Of the 61 animals, findings that contributed to death were identified in 33 (54%). Sixteen of the 61 whales (26%) were emaciated (poor body condition Figs 2A and 2B), 11 (18%) had lesions consistent with vessel strike (likelihood that vessel strike contributed to stranding was probable in six cases, and suspected in five cases, Figs 2C, 2D, and 2E), two (3%) had active entanglements, and one whale was entrapped. Nineteen cases (31%) had lesions consistent with killer whale interaction. Of these, five whales had lesions consistent with recent killer whale predation with the likelihood that killer whale predation contributed to mortality categorized as probable for two cases (Figs 2F and 2G), suspect for one case, and could not be determined in two cases (Fig 2H). The other 14 cases exhibited old/healed rake marks and killer whale predation was not considered to be a contributor to stranding. Lastly, a likely cause of death could not be identified in 28 whales (46%).

Table 6. Significant gross pathology and location of stranded gray whales documented in this paper (n = 61). CA=California, OR=Oregon, WA=Washington, BC=British Columbia, AK=Alaska.

**Cause of Death** ~~**Summary**~~	CA	OR	WA	BC	AK	**Totals**
Emaciation	2	1	13			**16**
Vessel strike	7		3	1		**11**
Active entanglements			2			**2**
Entrapment			1			**1**
Killer whale attack	1			1	1	**3**
Undetermined	X	X	X	X	X	**28**

Fig. 2: Examples of gross pathology findings. (A) Adult female gray whale category 1 emaciation showing loss of nuchal adipose tissue (white arrow) and severe epaxial muscle atrophy (black arrow); (B) Sub-adult male gray whale with blubber flensed from the right side to reveal severely atrophied hypaxial muscle (white arrow) and epaxial muscle (black arrow); (C) Sub-adult female gray whale with concave rostral deformity (arrow) caused by blunt force trauma; (D) Comminuted fracture of the rostral bones of whale C, arrow shows subjacent hypodermal contusion; (E) Adult male gray whale with exposed thoracic vertebrae showing transverse fractures of the dorsal vertebral processes (indicated by knife point) and muscular hemorrhage (arrow); (F) Gray whale calf predated by killer whales with loss of the tongue and soft tissue from the mandibles; (G) Flukes from the same calf showing acute epidermal lacerations consistent with killer whale tooth rakes (arrows); (H) Subadult gray whale pectoral flipper showing killer whale tooth rakes on the leading edge (arrow).

Nutritional condition

Using the standardized nutritional condition protocol (Appendix 1), there were sufficient data to assess the nutritional condition of 55 of 61 examined whales with 18 animals designated as emaciated, two from CA, one from OR, and 15 from WA; 28 animals identified as thin,

including nine from CA, 10 from WA, six from BC and three from AK (Table 7). For average animals, there were six from CA, three from BC and one from AK. For fat animals, there was one from CA and one from WA. Nutritional scores for five animals could not be determined: three from CA and two from BC. Factors that impeded the assessment of body condition included limited photographic documentation, post-mortem bloat, posture when beach cast (e.g. dorsal recumbency), substrate contour and other on-site variables. In five cases, the addition of microscopic evaluation of the blubber changed the nutritional status assigned grossly: two cases were changed from average to thin, two cases changed from thin to emaciated, and one case that could not be determined grossly was categorized as thin (Fig 3).

Table 7. Nutritional Score of stranded gray whales documented in this paper (n = 61). CA=California, OR=Oregon, WA=Washington, BC=British Columbia, AK=Alaska.

Location	Nutritional Score				
	Emaciation (Poor)	Thin (Fair)	Average (moderate)	Fat (Excellent)	CBD
CA	2	9	6	1	3
OR	1	0	0	0	0
WA	15	10	0	1	0
BC	0	5	2	0	2
AK	0	3	1	0	0
**Total**	**18**	**27**	**9**	**2**	**5**

Fig. 3 Blubber sample topography and histologic features. (A) Gray whale blubber full depth sample including the epidermis and panniculus muscle. White bars indicate sampling locations for the superficial, mid and deep blubber; (B1) Superficial blubber, adult female, , showing marked adipose atrophy (star) and relative abundance of connective tissue (arrowhead); (B2) Mid blubber layer from the same whale; (B3) deep blubber layer from the same whale; (C1) Superficial blubber layer, adult female, good body condition; (C2) Mid blubber layer and C3, deep blubber layer from the same well-conditioned whale.

Additional reported gross observations included diffuse salmon pink blubber (12 cases in WA and two in BC), as well as subcutaneous edema in two animals each stranded in WA, CA, and BC and one animal stranded in AK (Fig 4). The variation in color of the blubber was not

fully understood but was associated with more severely malnourished animals (seven were emaciated and seven were thin). Stomach contents were not consistently recorded, but wood chips, bark, eel grass, kelp, and some prey were identified within gastric contents (Fig 5).

Fig. 4: Grey whale blubber cut section. (A) Adult female in emaciated condition with pale pink blubber. (B) Adult female gray whale in thin condition stranded in Washington State with diffusely salmon pink blubber.

Fig. 5: Adult female gray whale ingesta. Stomach from an adult female gray whale opened to show content composed predominantly of plant based fibers and a few amphipod-like crustaceans.

For the 18 emaciated animals, the proximate cause of death was starvation in 16 of 18 (89%) cases, with no other lesions that would have contributed to antemortem morbidity or mortality detected. The other two emaciated whales had signs of blunt force trauma, which was the primary cause of death. Among the animals with thin and average nutritional condition, there were supervening processes which may have contributed to or resulted in the loss of the animal including evidence of blunt force trauma attributed to ship strike (n=8), suspect propeller wound (n=1), possible killer whale attack (n=2 probable), entrapment (n=1), gear entanglement (n=1), and historic inactive entanglement (n=1).

Vessel strike

Vessel strike was an important cause of mortality with gross findings of lethal blunt force trauma found in 11 of 61 cases (Table 7, Figs 2C, 2D, and 2E). Seven vessel strike cases were identified in CA, primarily in the San Francisco Bay area, three in WA and one in BC. Nutritional status varied for the vessel strike whales: two were emaciated, three were thin, five were average and one was fat. One additional whale had a suspected propeller wound on its flipper, but the likelihood that this contributed to stranding could not be determined; another whale had healed propeller scars that likely did not contribute to stranding.

Table 6. Nutritional Score of stranded gray whales documented in this paper (n = 61). CA=California, OR=Oregon, WA=Washington, BC=British Columbia, AK=Alaska.

Location	Nutritional Score				
	Emaciation (Poor)	Thin (Fair)	Average (moderate)	Fat (Excellent)	CBD
CA	2	9	6	1	3
OR	1	0	0	0	0
WA	15	10	0	1	0
BC	0	5	2	0	2
AK	0	3	1	0	0
**Total**	**18**	**27**	**9**	**2**	**5**

Fig. 2: Examples of gross pathology findings. (A) Adult female gray whale category 1 emaciation showing loss of nuchal adipose tissue (white arrow) and severe epaxial muscle atrophy (black arrow); (B) Sub-adult male gray whale with blubber flensed from the right side to reveal severely atrophied hypaxial muscle (white arrow) and epaxial muscle (black arrow); (C) Sub-adult female gray whale with concave rostral deformity (arrow) caused by blunt force trauma; (D) Comminuted fracture of the rostral bones of whale C, arrow shows subjacent hypodermal contusion; (E) Adult male gray whale with exposed thoracic vertebrae showing transverse fractures of the dorsal vertebral processes (indicated by knife point) and muscular hemorrhage (arrow); (F) Gray whale calf predated by killer whales with loss of the tongue and soft tissue from the mandibles; (G) Flukes from the same calf showing acute epidermal lacerations consistent with killer whale tooth rakes (arrows); (H) Subadult gray whale pectoral flipper showing killer whale tooth rakes on the leading edge (arrow).

Gear interactions and entrapment

One whale was reported entrapped in pilings and this contributed to its death. Five whales had evidence of active and inactive gear entanglements. Two stranded with active entanglements that contributed to death (one entanglement and drowning and one with a pectoral flipper amputation and gear present).

In three whales, the entanglements did not lead to death (one whale had a partially healed entanglement scar, and two had healed entanglement scars). These animals presented with superficial linear furrows, often with corrugated to well delineated margins circumferentially involving the axilla or fluke. At necropsy, the cutaneous defects were chronic with second intention healing and in one animal, there was focal secondary subcutaneous abscessation. However, advanced autolysis and position of the carcass at the time of necropsy may have

obscured lesions in other similarly affected whales. While these lesions were not associated with the stranding, they may have affected the health or nutritional state of the animal.

An adult male had been satellite tagged seven years prior to death and an appropriate granulation tissue response was associated with a fragment of the tag that remained in the blubber. While this whale was in thin body condition, the cause of death could not be determined [47].

Interactions with killer whales and sharks

There were 19 animals, eight adults and 11 subadults, with possible killer whale interactions characterized by active and inactive wounds. Fourteen whales had healed or more chronic rake marks predominantly on the tips of the fins, flukes, or more rarely along the rostrum that did not contribute to death. Five of these 19 carcasses had more acute active lesions, and in three of the whales these lesions likely contributed to death. This included one whale with bite wounds to the tongue with associated hemorrhage and edema of the lingual submucosa and muscularis (Figs 2F and 2G). Nutritional condition scores for the 5 of 19 cases with active killer whale lesions were one emaciated, two thin, one average and one that could not be determined. For the 14 of 19 animals with older evidence of killer whale interaction, 10 were emaciated, and four were thin. Also observed in three gray whales were smaller and more round to oval superficial cutaneous defects circumscribed by regular serrated margins, consistent with agonal or post-mortem shark bites.

Fig. 2: Examples of gross pathology findings. (A) Adult female gray whale category 1 emaciation showing loss of nuchal adipose tissue (white arrow) and severe epaxial muscle atrophy (black arrow); (B) Sub-adult male gray whale with blubber flensed from the right side to reveal severely atrophied hypaxial muscle (white arrow) and epaxial muscle (black arrow); (C) Sub-adult female gray whale with concave rostral deformity (arrow) caused by blunt force trauma; (D) Comminuted fracture of the rostral bones of whale C, arrow shows subjacent hypodermal contusion; (E) Adult male gray whale with exposed thoracic vertebrae showing transverse fractures of the dorsal vertebral processes (indicated by knife point) and muscular hemorrhage (arrow); (F) Gray whale calf predated by killer whales with loss of the tongue and soft tissue from the mandibles; (G) Flukes from the same calf showing acute epidermal

lacerations consistent with killer whale tooth rakes (arrows); (H) Subadult gray whale pectoral flipper showing killer whale tooth rakes on the leading edge (arrow).

In summary, gross post-mortem examination of 61 stranded animals identified the cause of death in 33 whales. This included 16 whales with emaciation as the only post mortem finding, 11 whales with evidence of vessel strike (including 2 that were also emaciated), 3 whales with pre-mortem killer whale attack (2 probable, 1 suspect), 2 entanglement cases, and 1 entrapment. There were 28 whales where cause of death could not be determined. There were distinct regional differences in stranding numbers, necropsy response efforts, demographics, and frequency of gross lesions and causes of death.

Histologic findings

Although post-mortem decomposition and bacterial overgrowth frequently hampered microscopic assessment of various tissues, especially internal viscera, histologic findings substantiated the gross observations of emaciation and trauma and revealed significant findings in 33 of 61 (54%) examined animals (Table 6). Microscopic findings were categorized by organ system and included skin and blubber (n=64 diagnoses), skeletal muscle (n=42), lung (n=11), gastrointestinal and liver (n=19), and miscellaneous (n=3) (Table 7). Multiple morphologic diagnoses in individual tissue sections accounted for a number greater than the 53 of 61 that were evaluated microscopically. The most common histologic findings included blubber atrophy (n=40, Fig 3B), hepatic hemosiderosis (n=15, Fig 6A), ulcerative or erosive dermatitis (n=14), muscle atrophy (n=10, Fig 6B), muscle edema (n=11), muscle necrosis (n=8), muscle degeneration (n=6, Fig 6C), muscle hemorrhage (n=5), and pulmonary edema (4), aspiration (n=3) and pneumonia (n=3). Histologic assessment of blubber showed a gradient in atrophy from the deepest to more superficial layers and featured prominent variation in the size and shape of adipocytes, and cytoplasmic condensation (atrophy or lipid depletion) with a disproportionate

abundance of stroma (Fig 3). Sporadic and incidental diagnoses included adrenal and myocardial hemorrhage, penile papilloma, myocellular sarcocystosis (Fig 6D), glossitis and lingual mucosal inclusions (Figs 6E and 6F), gastric erosions and enteric cestodiasis (Table 8). Due to logistical challenges at the time of necropsy and varying states of tissue decomposition, not all organs or tissues were systematically examined or collected, so histologic lesions were likely underrepresented.

Table 7. Significant gross pathology and location of stranded gray whales documented in this paper (n = 61). CA=California, OR=Oregon, WA=Washington, BC=British Columbia, AK=Alaska.

**Cause of Death Summary**	CA	OR	WA	BC	AK	**Totals**
Emaciation	2	1	13			**16**
Vessel strike	7		3	1		**11**
Active entanglements			2			**2**
Entrapment			1			**1**
Killer whale attack	1			1	1	**3**
Undetermined	X	X	X	X	X	**28**

Table 8. Gross and microscopic lesions identified in stranded gray whale tissues from 53 of 61 whales. The data were generated by reviewing histopathology reports and compiling frequency of morphologic diagnoses. CA=California, OR=Oregon, WA=Washington, BC=British Columbia, AK=Alaska.

**Morphologic diagnoses**	CA	OR	WA	BC	AK	**Totals**
**Skin and Blubber**						
Dermatitis	3	0	5	3	1	**12**
Ulcerative or erosive dermatitis	1	0	6	6	2	**15**
Fat necrosis	0	0	0	0	0	**0**
Blubber atrophy	13	0	19	7	1	**40**
**Skeletal Muscle**						
Muscle atrophy	7	0	0	3	0	**10**
Muscle edema	6	0	1	4	0	**11**
Muscle necrosis	4	0	2	1	1	**8**
Muscle degeneration	3	0	0	2	1	**6**
Muscle hemorrhage	5	0	0	0	0	**5**
Muscle Sarcocysts	1	0	0	0	1	**2**
**Lung**						
Pulmonary edema	1	0	1	2	0	**4**

Pulmonary aspiration	1	0	1	1	0	**3**
Pneumonia	1	0	0	1	1	**3**
Myocardial hemorrhage	1	0	0	0	0	**1**
**Gastrointestinal and Liver**						
Gastric erosions	0	0	1	0	0	**1**
Enteric cestodes	0	0	1	0	0	**1**
Tongue cytoplasmic inclusions	1	0	0	0	0	**1**
Liver hemosiderosis	2	0	11	2	0	**15**
Liver atrophy	0	0	0	1	0	**1**
**Miscellaneous**						
Adrenal hemorrhage	1	0	0	0	0	**1**
Penile papilloma	0	0	1	0	0	**1**
Liver inclusions	1	0	0	0	0	**1**

Fig. 3 (A) Gray whale blubber full depth sample including the epidermis and panniculus muscle. White bars indicate sampling locations for the superficial, mid and deep blubber; (B1) Superficial blubber, adult female, , showing marked adipose atrophy (star) and relative abundance of connective tissue (arrowhead); (B2) Mid blubber layer from the same whale; (B3) deep blubber layer from the same whale; (C1) Superficial blubber layer, adult female, good body condition; (C2) Mid blubber layer and C3, deep blubber layer from the same well-conditioned whale.

Fig. 6 (A) Representative histopathology of stranded gray whales. Liver, adult female gray whale, emaciated body condition, marked diffuse hemosiderosis, H&E stain, x400 magnification, bar=20µm; (B) Skeletal muscle, subadult male gray whale, thin body condition, moderate diffuse myocyte atrophy. H&E stain, x200 magnification, bar=50µm; (C) Skeletal muscle, adult female gray whale, emaciated, severe diffuse myocyte degeneration. H&E stain, x200 magnification, bar=50µm; (D) Skeletal muscle, adult male gray whale, thin body condition, moderate diffuse myocyte atrophy and protozoal tissue cyst, *Sarcocystis* spp. presumptive. H&E stain, x200 magnification, bar=50µm; (E) Tongue, gray whale calf, glossitis with epidermal hyperkeratosis, hydropic change and necrosis of acanthocytes with neutrophilic infiltration (intraepithelial pustules, blue arrow head) and intralesional bacterial colonies (black arrow). H&E stain, x100 magnification, bar=100µm; (F) Tongue, gray whale calf, glossitis as in E, acanthocytes with intracytoplasmic and intranuclear eosinophilic inclusion bodies (arrows). H&E stain, x200 magnification, bar=50µm.

Discussion

In December of 2018, increased numbers of dead gray whales were first reported stranded in Mexico, with subsequent increases in mortality in 2019 over the northward migration

spanning from CA to OR, WA, BC, and ultimately, AK. An UME was declared by the NMFS in 2019, which mobilized and coordinated transboundary stranding response efforts, shared expertise, and facilitated communication between regional response programs and the public.

As with the 1999-2000 gray whale UME investigation, emaciation was identified as a frequent cause of morbidity or mortality in the subset of whales examined in this current UME. Definitions of emaciation for long-distance migratory whales that fast for substantial lengths of time in the annual life cycle are complicated, and it became apparent that different definitions were applied in different locations. Further, blubber depth, while easily measured in accessible whales, is not a sensitive measure of nutritional status in gray whales [25]. Because of a lack of available quantitative tests to assess nutritional condition, especially given the variability in carcass presentation and accessibility, definitions of nutritional states were developed during this investigation and standardized across all stranding response networks to ensure consistent interpretation of post-mortem body condition (Appendix 1). Despite standardization, further work is needed to characterize the nutritional status of dead gray whales. Microscopic evaluation of blubber and other tissues was found to be of value in this investigation and could be standardized for future protocols; and validation and measurement of biomarkers may also be helpful in the future. Interpretation of nutritional status from stranded whales can be further improved by the integration of ecologic and environmental parameters as well body condition indices from live gray whales; for example, [16] used photogrammetry to detect a decrease in body condition in 2018 compared to previous years. In addition, photogrammetry was used to describe reduced body condition in live gray whales in Mexico in 2018 and 2019 compared to 2017 [48]. Both of these studies coincide with our findings since 2019 is the year when we detected the greatest number of emaciated whales (n=14).

As gray whales are usually thin during the northward migration, it is difficult to assign thin or emaciated body condition as a proximate or immediate cause of death; however, in more

severely emaciated animals (code 1 nutritional status, Fig 2A and 2B), malnutrition would presumably have contributed to antemortem morbidity, dehydration, loss of buoyancy and eventual live or dead stranding [25]. Supervening ectoparasite (Cyamidae, whale lice) infestations (Fig 7) and secondary cutaneous ulcers and erosions were consistently noted in emaciated animals in this case series and the loss of buoyancy in more extreme cases of malnutrition may have resulted in carcasses lost at sea, rather than potentially submerged, refloated, beach cast, and thus available for necropsy [49].

Fig. 7: Emaciated subadult male gray whale with ectoparasitic copepods. View of genital and perineal area showing massive infestation with cyamids (whale lice). Inset shows closer view of cyamids surrounding attached cirripedes (barnacles) on the skin surface.

Important sequelae to loss of blubber mass or insufficient caloric consumption in the summer feeding grounds may also include infertility (anestrous), early embryonic loss and fetal resorption, abortion, premature birth, death of dependent calves, live stranding, a reduced ability to refloat and possible death due to asphyxiation or a compartmental-type syndrome [50]. In the longer term, malnourished females may experience reduced fecundity, prolonged inter-calving interval, or decreased life longevity, which can lead to low calf recruitment as documented in the recent calf production surveys [21]. Extralimital foraging efforts on the northern migration may result in greater use of coastal bays and sounds on the west coast (e.g. San Francisco Bay, CA, Puget Sound, WA and Port of Los Angeles, CA); increased time spent in shipping lanes may increase the risk of vessel strike, as well as increasing exposure to environmental sound disturbances and possible disorientation. While our numbers were too small for statistical analysis, there did not appear to be an association between suboptimal nutritional status and increased propensity for traumatic injury.

In many cases, the physiologic demands associated with prolonged migration, parturition, lactation, and reproduction may account for the decline in nutritional condition; however, the

increased proportion of emaciated whales documented in WA relative to Mexico, CA, and OR suggests metabolic consequences due to depleted fat stores, prolonged inappetence, possible anorexia and other factors during the northward migrations along the west coast. Unidentified environmental or infectious factors may have also contributed to the higher incidence of emaciated animals in this region. Dietary factors may include relative abundance and nutritive quality of prey species [15] or the population of gray whales may be approaching carrying capacity. In multiple animals, wood chips, bark, eel grass, kelp and inorganic debris were identified within gastric contents suggesting foraging attempts in sub-optimal habitat. Based on available data and the lack of historic use of a standard nutritional condition protocol, it is difficult to determine whether stranded whales with severe emaciation represent a novel process unique to this UME and the 1999-2000 UME or if these animals represent typical nutritional status during this phase of the northbound migration in non-UME years as well. More work would be needed to assess historic cases using the nutritional condition protocol developed for this UME for comparison.

The observation of a gradation in atrophy of adipose through the blubber layer suggests that greater effort is required to assess this condition more consistently and to factor it into the nutritional condition score matrix in the future (Appendix 1). Advanced autolysis in many cases precluded evaluation of possible endogenous pigments, such as bile or exogenous compounds, such as carotenoids. The accumulation of iron within the examined livers and atrophy of skeletal muscle in animals stranded in WA and CA substantiated the gross diagnosis of thin or emaciated animals. Myocellular atrophy and atrophy of adipose tissue are consistent with the gross observations of thin or emaciated animals. Hepatocellular hemosiderosis can be associated with malnutrition, acute infections (with iron sequestration), chronic inflammation, a maladaptive type syndrome, excessive dietary iron consumption, and other factors. In addition, more work is

needed to understand the microscopic blubber changes with migration and fasting and with decomposition.

In this case series it is difficult to infer a direct association between the salmon discoloration of the blubber observed in WA whales and malnutrition and hepatocellular hemosiderosis. There were 14 animals in suboptimal nutritional condition with discoloration of the blubber; 7 of the 14 cases were thin and 7 of 14 were emaciated and hepatocellular hemosiderosis was reported in 7 animals. The salmon discoloration was distinct from jaundice and was most likely due to exogenous pigment deposition, possibly related to prey shifts and consumption of a diet richer in carotenoids. The subcutaneous edema noted in a few cases of discolored blubber may be secondary to hypoproteinemia, localized trauma, or impaired vascular perfusion as a consequence of live stranding.

Vessel strikes were important causes of mortality in this case series. In the 1999-2000 UME, only one confirmed case of vessel strike was documented; however, it is important to note that only three stranded animals were necropsied during the previous UME [25]. Most of the stranded gray whales with gross lesions consistent with vessel strike (Fig 2) were recovered near the shipping channels leading into San Francisco Bay, CA and Puget Sound, WA, which are areas of high relative risk for gray whale vessel strike [22]. Three whales in more remote areas (BC and AK) also had injuries consistent with ship strikes outside of large shipping channels. Some known vessel strike cases reported during the UME were not included in this case study because carcasses were not examined, or due to advanced decomposition at the time they were accessed, as such, the vessel strike numbers reported here are an underestimate. Further analysis of all the vessel strike cases during UME and non-UME years would determine if there has been a change in the rate of vessel strike mortality.

Scars from killer whale attacks are quite common in a variety of large whale species. In a comparison study among blue, humpback, and gray whales in the eastern north Pacific, gray

whales had the highest incidence of killer whale rake marks in live animals with 42% seen in photographs [51]. In more severely affected carcasses, complete loss of the soft tissues (mucocutaneous junction and mucosa) from either or both mandibles and separation of the mandibular symphysis have been reported [45]. In addition, [44] noted the following in whales predated by killer whales: Segmental detachment or stripping of segments of the skin and blubber from the underlying fibroelastic sheath with smooth abrupt linear or curvilinear margins and more rarely, focal to segmental abdominal perforations with abrupt irregular margins and visceral exteriorization. In presumptive non-lethal attacks, there were associated superficial linear to curvilinear fenestrated cutaneous furrows or rake marks on the appendages, and variably sized elliptical to crescentic defects of the caudal limits of the pectoral fins and flukes that were attributed to bite wounds. Development of the case definition for killer whale predation as a cause of death was an important step towards evaluating these cases consistently throughout the gray whale range. Hopefully these criteria can differentiate vessel strike from killer whale predation in AK, where increased vessel traffic through the Bering Strait and pods of transient killer whales that predate gray whale calves occur simultaneously (C. Matkin, pers comm). Detailed external examinations of the OR gray whales not described in this paper, revealed substantial evidence of killer whale interactions. Future efforts are underway to evaluate all the gray whale UME cases for signs of killer whale interactions which may illuminate previously undescribed patterns in killer whale behavior.

Carcass decomposition severely limited the ability to conduct comprehensive necropsies on whales with full sampling for histologic examination to rule out primary or secondary involvement of infectious disease, toxins or other disease processes in this UME [26]. Incidental and solitary cases of myocellular sarcocystosis, penile papilloma, pneumonia, enteric cestodiasis and gastric erosions were interesting and valuable observations (Table 8), but did not appear to contribute substantially to the individual health of stranded animals. One of the two calves

examined, live-stranded in CA and died spontaneously after several hours on a beach. At necropsy, there were no significant gross lesions but histopathology revealed mild acute sub-endocardial and adrenal cortical hemorrhage consistent with agonal release of catecholamines (as described in [52]). These lesions may also be associated with septic or hypovolemic shock and other processes but there was no histological evidence for systemic infection. An additional histologic finding was mild bacterial glossitis with cytoplasmic inclusions in the lingual mucosa. Follow up transmission electron microscopy revealed that the cytoplasmic inclusions were composed of keratin filaments and glycogen and were considered an incidental finding.

Due to inclement weather conditions, the remoteness or inaccessibility of many strandings, human safety concerns, and level of decomposition, internal examinations are not pursued on all strandings, however the gross and microscopic findings we documented did not suggest that infectious agents were a cause of death or stranding during this UME. Based on the lack of inflammation and degree of pulmonary edema, the aspirated debris in lung sections in a few whales was most likely agonal and associated with live stranding. As mortality due to infectious disease can be associated with complex interactions with environmental factors, poor body condition, and etiologic agent, testing of existing samples from this UME for pathogens and biotoxins is in progress, and prospective investigations on fresh carcasses should focus on collecting appropriate samples for more advanced analyses.

The northward migration of gray whales along the US West Coast in spring is an annual event that for decades has been documented and reported by generations of biologists and naturalists. Many portions of the coast are populated and well-traveled, so that many large cetacean strandings are likely observed and reported. In contrast, in Alaska, carcass discovery is more dependent upon targeted aerial surveys. For this reason, it is unlikely that the large peaks in mortality recorded in 1999-2000 and 2019-2021 are due to increased observational effort. Beach cast and near-shore floating animals represent a small percentage of the overall mortality of large

whale populations [53-55]. Factors that impact the stranding event include that the whale dies close enough to shore to eventually strand, that the oceanographic conditions propel the animal to come ashore, that the stranded animal is detected, and that a response team is able to access the carcass. Because of the extensive nature of the North American coastline, the stark differences in population density, the distribution of response groups, and the often remote stranding locations of whales, this sample of gray whale strandings is an underestimate of the mortality during this period. Data collection from this study's subset of whales was further limited by decomposition and logistics. The contribution of travel restrictions and the lack of public shore usage associated with the COVID-19 pandemic is unknown; however, in some regions along the coast, responses were curtailed or restricted.

While malnutrition, affecting all age classes, appears to have played a role in gray whale morbidity and mortality in 1999-2000 and 2019-2021, the factors driving this, including environmental influences and concurrent health issues, and their temporal relationships to increases in mortality, are unclear. In particular, the impact of oceanic thermal anomalies in the North Pacific [56], unprecedented loss of winter sea ice and extreme summer sea surface temperatures in the Bering and Chukchi marine shelf ecosystems between 2017 and 2019, and changes in infaunal, benthic and pelagic prey abundance and nutritive quality to the morbidity and mortality of gray whales require further research [14-16]. The current data and review of the 2019-2021 strandings, as well as comparison with the epidemiologic and necropsy findings from the 1999-2000 UME, support the hypothesis that these events are likely recurring and transitory. Finally, the roles of infectious disease, or intoxication (e.g., biotoxins), as examples of stochastic mortality [57], cannot be ruled out by the current investigation and additional testing of samples will be pursued in the future. The review of gray whale health and disease findings from this investigation, coupled with a recent overview of gray whale pathology literature [26], will better inform future studies of stranded animals.

Gray whales continue to strand, and sampling is ongoing with more data and testing to be conducted on archived samples. The data presented here are a small contribution to the overall UME investigation and other teams are working on evaluating environmental and population changes that may be impacting the whales. A better understanding of the manifestations and mechanisms involved in atrophy of blubber during the migration will assist in the determination of normal physiologic atrophy versus malnutrition for this migratory species. Ongoing efforts to document changes in the nutritional status and behavior of free ranging animals, calf recruitment, and foraging behavior will further contribute to understanding the recurrent stranding events of the Eastern North Pacific gray whale population.

In conclusion, the declaration of the UME was invaluable in establishing effective channels of communication and developing and standardizing response protocols across stranding response networks, as well as regional, national, and international governmental partners and agencies which enhanced surveillance and reporting of floating and stranded dead gray whales. The preliminary findings from the subset of 61 whales presented here have found malnutrition, vessel strikes, killer whale predation, gear entanglement and entrapment as causes of death. The necropsy findings of malnutrition support the findings of poor body condition seen in live free ranging whales and low calf recruitment during the UME period suggesting that environmental drivers are impacting the Eastern North Pacific gray whale population and contributing to some of the increased strandings.

Ethics Statement

The gray whales included in this study were reported either floating dead in near shore waters and towed ashore, or beach cast. Morphometrics and necropsies were conducted according to conventional protocols with the authorization of the National Marine Fisheries Service Marine

Mammal Health and Stranding Response Program (US) and Fisheries and Oceans, Canada with appropriate supporting documentation to collect, retain and transport tissues. In this case series, tissue collection and morphometrics were compiled only on dead animals. No live whales were euthanized.

Acknowledgements

All marine mammal stranding activities in the US were conducted under authorization by the NMFS through Stranding Agreements issued to the Marine Mammal Stranding Network Organizations involved in this UME. We acknowledge the assistance of numerous staff and volunteers within the U.S. Marine Mammal Stranding Networks in CA, OR, WA and AK and especially The Marine Mammal Center (TMMC) and the California Academy of Sciences with specific mention of Dr. C. Field, E. Whitmer and J. Isbell (TMMC). We thank C. Matkin for comments regarding killer predation of gray whales in AK and Dr. S. Moore for invaluable insights. The First Nations communities, Department of Fisheries and Oceans, Fisheries Officers of BC and staff at the BC Animal Health Center, Abbotsford, BC are gratefully acknowledged. Funds for many stranding responses were provided by the Prescott Grants and the Department of Fisheries and Oceans. Tissue collection and processing was conducted under NMFS Stranding Agreements or the Marine Mammal Stranding Response Program Permit number 18768, issued under Section 104 (16 U.S.C. 1374) of the Marine Mammal Protection Act. In MX, the gray whale stranding field work was conducted under the permit from the Secretaría del medio Ambiente y Recursos Naturales No. SGPA/DGVS/013210/18. We thank Steven Swartz, Sergio Martínez A., Lorena Viloria G. and the personnel of Laguna San Ignacio Ecosystem Science Program, PRIMMA-UABCS and the Reserva de la Biósfera El Vizcaino of CONANP. The

scientific results and conclusions, as well as any views or opinions expressed herein, are those of the authors and do not necessarily reflect the views of NOAA.

Author Contributions Statement

SR, PD, DG, JH, KB-H, JC, PC, KD, DD'A, DD, MF, FMDG, BH, DML, TL, JUR, JR, KS, JG, JV, MM and BC participated in necropsies, compiling morphometrics and tissue disposition and archiving. Histopathology was performed by PD, MG, KB-H, MM and SR. DG, JH, KB-H, JC, PC, KD, DD'A, MF, FG, BH, DL, JUR, JR, KW, JV, JG, BC and DF compiled and uploaded data. DG, SR, PD, DG, TL, KS, and BC compiled and analyzed data with assistance from all co-authors. All co-authors participated in drafting, reviewing and revising the manuscript. KW, TR, PC, TL, JG, JV, and DF administer regional and national stranding response programs. All coauthors approve the final submission.

Conflict of Interest Statement

MG founded the Northwest ZooPath and KB-H is the proprietor of the Alaska Veterinary Pathology Services. Otherwise, the authors declare that the research was conducted in the absence of any commercial or financial relationships that could be construed as a potential conflict of interest.

Data availability

The Level A data for U.S. whales is available to be requested on the NMFS website and other relevant data may be obtained after the close of the UME by request to the NMFS regional stranding coordinators, corresponding authors, or data contributors.

References

1. Lang AR, Weller DW, Burdin AM, Robertson K, Sychenko O, Urbán RJ, et al. Population structure of North Pacific gray whales in light of trans-Pacific movements. Mar Mamm Sci. 2022; 38:433-468.
2. Harris J, Calambokidis J, Perez A, Mahoney P J. Recent trends in the abundance of seasonal gray whales (*Eschrichtius robustus*) in the Pacific Northwest, 1996-2020. 2022; AFSC Processed Rep. 2022-05, 22 pp.
3. Weller DW, Klimek A, Bradford AL, Calambokidis J, Lang AR, Gisborne B, et al. Movements of gray whales between the western and eastern North Pacific. Endanger Species Res. 2012; 18:193-199.
4. Calambokidis J, Laake J, Pérez A. Updated analysis of abundance and population structure of seasonal gray whales in the Pacific Northwest, 1996-2015. 2017; Paper SC/A17/GW/05 presented to the International Whaling Commission.
5. Mate BR, Ilyashenko VY, Bradford AL, Vertyankin VV, Tsidulko GA, Rozhnov VV, et al. Critically endangered western gray whales migrate to the eastern North Pacific. Biol Lett. 2015; 11(4), 20150071. doi:10.1098/rsbl.2015.0071.
6. Durban J, Weller D, Lang A, Perryman W. Estimating gray whale abundance from shore-based counts using a multilevel Bayesian model. J Cetacean Res Manag. 2015; 15: 61-68.
7. Durban J, Weller DW, Perryman WL. Gray whale abundance estimates from shore-based counts off California in 2014/2015 and 2015/2016. 2017. Paper SC/A17/GW/06 presented to the International Whaling Commission.
8. Carretta, JV, Forney KA, Oleson EM, Weller DW, Lang AR, Baker J, et al. U.S. Pacific marine mammal stock assessments: 2018. U.S. Department of Commerce, NOAA Technical Memorandum. 2019; NMFS-SWFSC-617.

9. Gavrilchuk K, Doniol-Valcroze T. Recovery potential assessment for the grey whale (*Eschrichtius robustus*): Pacific Coast Feeding Group and Western Pacific Population in Canadian Waters. DFO Can Sci Advis Sec Res Doc. 2021; 2021/020. iv + 52 p.

10. Scammon, CM. The Marine Mammals of the North-Western Coast of North America, Described and Illustrated: Together with an Account of The American Whale-Fishery. John H. Carmany and Company. San Francisco. 1874.

11. Bruniche-Olsen A, Westerman R, Kazmierczyk Z, Vertyankin VV, Godard-Codding C, Bickham JW, et al. The inference of gray whale (*Eschrichtius robustus*) historical population attributes from whole-genome sequences. BCM Evol Biol. 2018; 18(1):87. doi: 10.1186/s12862-018-1204-3

12. Alter SE, Newsome SD, Palumbi SR. Pre-whaling genetic diversity and population ecology in eastern Pacific gray whales: insights from ancient DNA and stable isotopes. PLoS One. 2012; 7(5):e35039.

13. Eguchi, T, Lang, AR, Weller, DW. Abundance and migratory phenology of eastern North Pacific gray whales 2021/2022. U.S. Department of Commerce, NOAA Technical Memorandum NMFS-SWFSC-668. 2022. https://doi.org/10.25923/x88y-8p07

14. Huntington H, Danielson S, Wiese F, Baker M, Boveng P, Citta J, et al. Evidence suggests potential transformation of the Pacific Arctic ecosystem is underway. Nat Clim Chang. 2020; doi.org/10.1038/s41558-020-0695-2.

15. Moore SE, Clarke JT, Okkonen SR, Grebmeier JM, Berchok CL, Stafford KM. Changes in gray whale phenology and distribution related to prey variability and ocean biophysics in the north Bering and eastern Chukchi seas. PLos ONE. 2022; 17(4):e0265934.

16. Soledade Lemos L, Burnett JD, Chandler TE, Sumich JL, Torres LG. Intra- and inter-annual variation in gray whale body condition on a foraging ground. Ecosphere. 2020; 11(4): doi.org/10.1002/ecs2.3094.

17. Perryman WL, Joyce T, Weller DW, Durban JW. Environmental factors influencing eastern North Pacific gray whale calf production 1994–2016. Mar Mamm Sci. 2021; 37(2), 448-462.

18. Perryman WL, Donahue MA, Perkins PC, Reilly SB. Gray whale calf production 1994-2000: Are observed fluctuations related to changes in seasonal ice cover? Mar Mamm Sci. 2002; 18, 121-144.

19. Gailey G, Sychenko O, Tyurneva O, Yakovlev Y, Vertyankin V, van der Wolf P, et al. Effects of sea ice on growth rates of an endangered population of gray whales. Sci Rep. 2020; 10:1553. pmid:32005947.

20. Stewart JD, Weller DW. Abundance of Eastern North Pacific gray whales 2-19/2020. U.S. Dept. Comm., NOAA Technical Memorandum. 2021; NMFS-SWFSC-639.

21. Eguchi T, Lang AR, Weller DW. Eastern North Pacific Gray Whale Calf Production 1994-2022. U.S. Department of Commerce, NOAA Technical Memorandum. 2022. NMFS-SWFSC-667. https://doi.org/10.25923/4g6h-9129.

22. Silber GK, Weller DW, Reeves RR, Adams JD, Moore TJ. Co-occurrence of gray whales and vessel traffic in the North Pacific Ocean. Endanger Species Res. 2021; 44, 177-201.

23. Scordino J, Carretta J, Cottrell P, Greenman J, Savage K, Scordino J, et al. Ship strikes and entanglements of gray whales in the North Pacific Ocean, 1924-2015. 2017. Paper SC/67A/HIM/06 presented at the 18th meeting of the Western Gray Whale Advisory Panel.

24. Moore, MJ, van der Hoop, JM. The painful side of trap and fixed net fisheries: chronic entanglement of large whales. J of Mar Biol. 2012; Article ID 230653, doi:10.1155/2012/230653.

25. Gulland FMD, Pérez-Cortes M, Urgán J, Rojas-Bracho RL, Ylitalo G, Weir J, et al. Eastern North Pacific gray whale (*Eschrichtius robustus*) unusual mortality events, 1999-2000. U.S. Dept of Commerce, NOAA tech. Memo. 2005; NMFS-AFSC-150, 33 pp.

26. Stimmelmayr R, Gulland FMD. Gray Whale (*Eschrichtius robustus*) health and disease: review and future directions. Front in Mar Sci. 2020; 7:588820.

27. Rice DW, Wolman AA. The life history of the gray whale, (*Eschrichtius robustus*). American Society of Mammologists. 1971; Special Publication 3. 142pp.

28. Dailey M, Gulland FMD, Lowenstein L, Silvagni P, Howard D. Prey, parasites and pathology associated with the mortality of a juvenile gray whale (*Eschrichtius robustus*) stranded along the northern California coast. Dis Aquat Organ. 2000; 42, 111–117. doi: 10.3354/dao042111.

29. Benson SR, Croll DA, Marinovic BB, Chavez FP, Harvey JT. Changes in the cetacean assemblage of a coastal upwelling ecosystem during El Niño 1997–98 and La Niña 1999. Prog Oceanogr. 2002; 54(1): 279–291.

30. Caraveo-Patiño J, Soto LA. Stable carbon isotope ratios for the gray whale (*Eschrichtius robustus*) in the breeding grounds of Baja California Sur, Mexico. Hydrobiologia. 2005; 539(1): 99–104. doi:10.1007/s10750-004-3370-0.

31. Moore SE, Urban R, Perryman W, Gulland FMD, Perez-Cortes MH, Wade P, et al. Are gray whales hitting “K” hard? 2001 Mar Mamm Sci. 2001; 71: 954-958.

32. Moore SE, Grebmeier JM, Davies JR. Gray whale distribution relative to forage habitat in the northern Bering Sea: current conditions and retrospective summary. Can J of Zool. 2003; 81(4): 734-742.

33. Punt AE, Wade PR. Population status of the eastern North Pacific stock of gray whales in 2009. U.S. Dep. Commer., NOAA Tech Memo. 2010; NMFS-AFSC-207, 43 p.

34. LeBoeuf BJ, Perez Cortes MH, Urban RJ, Mate BR, Ollervides UF. High gray whale mortality and low recruitment in 1999: potential causes and implications. J Cetacean Res Manag. 2000; 2:85-99

35. Sumich JL. Growth in young gray whales (*Eschrichtius robustus*). Mar Mamm Sci. 1986; 2(2): 145-152.

36. Sumich JL, Goff T, Perryman WL. Growth of two captive gray whales (*Eschrichtius robustus*). Aquat Mamm. 2001; 27(3): 231-233.

37. Sumich JL, Blokin SA, Tiupeleyev PA. Revised estimates of foetal and post-natal growth in young gray whales (*Eschrichtius robustus*). J Cetacean Res Manage. 2013; 13(2): 89-96.

38. Bradford AL, Weller DW, Burdin AM, Brownell RL. Using barnacle and pigmentation characteristics to identify gray whale calves on their feeding grounds. Mar Mamm Sci. 2011; 27(3):644-651.

39. Agbayani S, Fortune SME, Trites AW. Growth and development of North Pacific gray whales (*Eschrichtius robustus*). J Mammal. 2020; 101(3):742-754.

40. Cottrell P, Cottrell B, Dowdall T, Hoyland Al, Hoyland AS, Raverty S. Recovery of a mid-gestational gray whale (*Eschrichtius robustus*) fetus near Tofino, British Columbia. Aquat Mamm. 2022; 48(6), 626-633, DOI 10.1578/AM.48.6.2022.626.

41. Geraci, J, Lounsbury, V 2005. Evaluating a carcass. In: Geraci J, Lounsbury VJ, editors. Marine mammals ashore: a field guide for strandings. 2nd ed, Baltimore: National Aquarium; 2005. Pp. 182-194.

42. Raverty S, Duignan PJ, Jepson P, Morel M. Necropsy procedures and sampling for marine mammal gross necropsy. In Gulland FMD, Dierauf LA, Whitman K.L, editors. The CRC handbook of marine mammal medicine, 3rd ed. Boca Rotan: CRC Press; 2018. pp. 249-266.

43. Moore-Touhey K, Barco, SG. Handbook for recognizing, evaluating, and documenting human interaction in stranded cetaceans and pinnipeds. NOAA-TM-NMFS-SWFSC. 2013; 510; NOAA technical memorandum NMFS; URL: https://repository.library.noaa.gov/view/noaa/4429

44. Lowry L, Nelson R, Frost K. Observations of killer whales, *Orcinus orca*, in western Alaska: sightings, strandings, and predation on other marine mammals. Can Field Nat 1987; 101:6–12.

45. Melnikov VV, Zagrebin IA. Killer whale predation in coastal water of the Chukotka Peninsula. Mar Mammal Sci. 2005; 21:550–556. https://doi.org/10.1111/j.1748-7692.2005.tb01248.x.

46. Barrett-Lennard L, Matkin C, Durban J, Saulitis E, Elfret, D. Predation on gray whales and prolonged feeding on submerged carcasses by transient killer whales in Unimak Island, Alaska. Mar Ecol Prog Ser. 2011; 421:229-241.

47. Goley PD., Calambokidis J, Duignan P, Gulland FMD, Halaska B, Lui A, Martinez M, and Mate B. Observations of tissue healing around a consolidated radio tag in a Pacific coast feeding group gray whale (*Eschrichtius robustus*). Mar Mamm Sci. Forthcoming

48. Christiansen F, Rodriguez-Gonzalez F, Martinez-Aguilar S, Urban J, Swartz S, Warick H, et al. Poor body condition associated with an unusual mortality event in gray whales. Mar Ecol Prog Ser. 2021; 658: 237-252 https://doi.org/10.3354/meps13585.

49. Moore M, Mitchell G, Rowles T, Early D. Dead cetacean? beach, bloat, float, sink. Front in Mar Sci. 2020; 7, 333 doi: 10.3389/fmars.2020.00333.

50. Herráez P, Espinosa De Los Monteros A, Fernandez A, Edwards J, Sacchini S, Sierra E. Capture myopathy in live-stranded cetaceans. Vet J. 2013; 196(2): 181-188.

51. Corsi E, Calambokidis J, Flynn KR, Steiger GH. 2021. Killer Whale Predatory Scarring on Mysticetes: A comparison of rake marks among blue, humpback, and gray whales in the Eastern North Pacific. Mar Mamm Sci. 2021; 38(1): 223-234. doi: 10.1111/mms.12863.

52. Harruff RC. Subendocardial hemorrhages in forensic pathology autopsies. Am J Forensic Med and Path. 1993; 14(4), 284-288.

53. Carretta JV, Danil K, Chivers S, Weller DW, Janiger DS, Berman-Kowalewski M, et al. 2016. Recovery rates of bottlenose dolphin (*Tursiops truncatus*) carcasses estimated from stranding and survival rate data. Mar Mamm Sci. 2016; 32(1):349-362.

54. Wells RS, Allen JB, Lovewell G, Gorzelany J, Delynn RE, Fauquier DA, Barros NB. Carcass-recovery rates for resident bottlenose dolphins in Sarasota Bay, Florida. Mar Mamm Sci. 2015; 31:355–368.

55. Williams R, Gero S, Bejder L, Calambokidis J, Krais, SD, Lusseau, D, et al. Underestimating the damage: Interpreting cetacean carcass recoveries in the context of the Deepwater Horizon/BP incident. Conserv Lett. 2011; 4:228-233.

56. Cornwall W. A new blob menaces Pacific ecosystems. Science. 2019; 365 (6459): 1233

57. Lefebvre KA, Quakenbush L, Frame E, Burek Huntington K, Sheffield G, Stimmelmayr R, et al. Prevalence of algal toxins in Alaskan marine mammals foraging in a changing arctic and subarctic environment. Harmful Algae. 2016; 55:13–24.

Figure

Click here to access/download;Figure;Fig1.tiff.tif

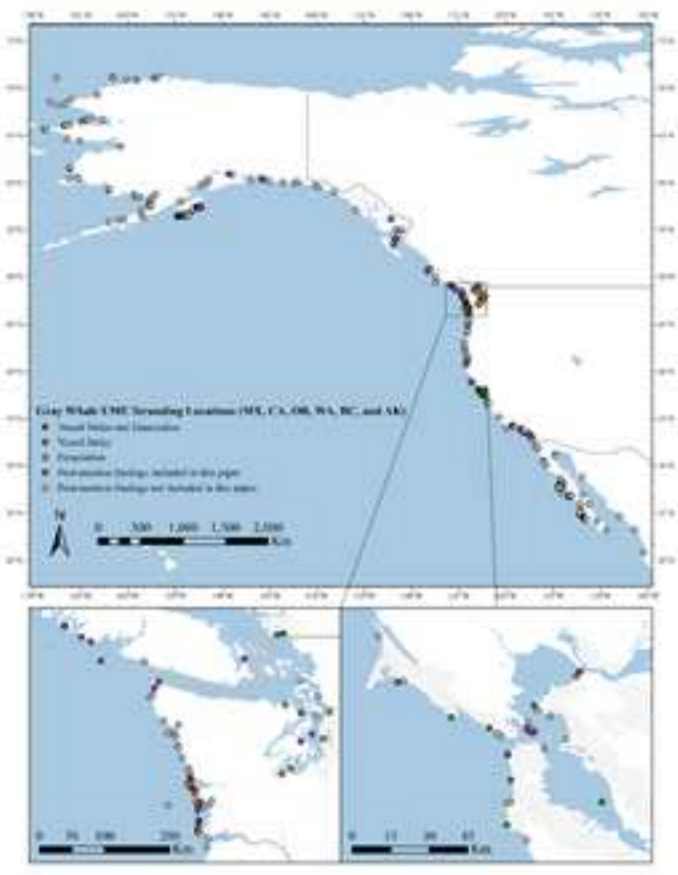

 Click here to access/download;Figure;Fig2.tiff.tif

Fig. 2

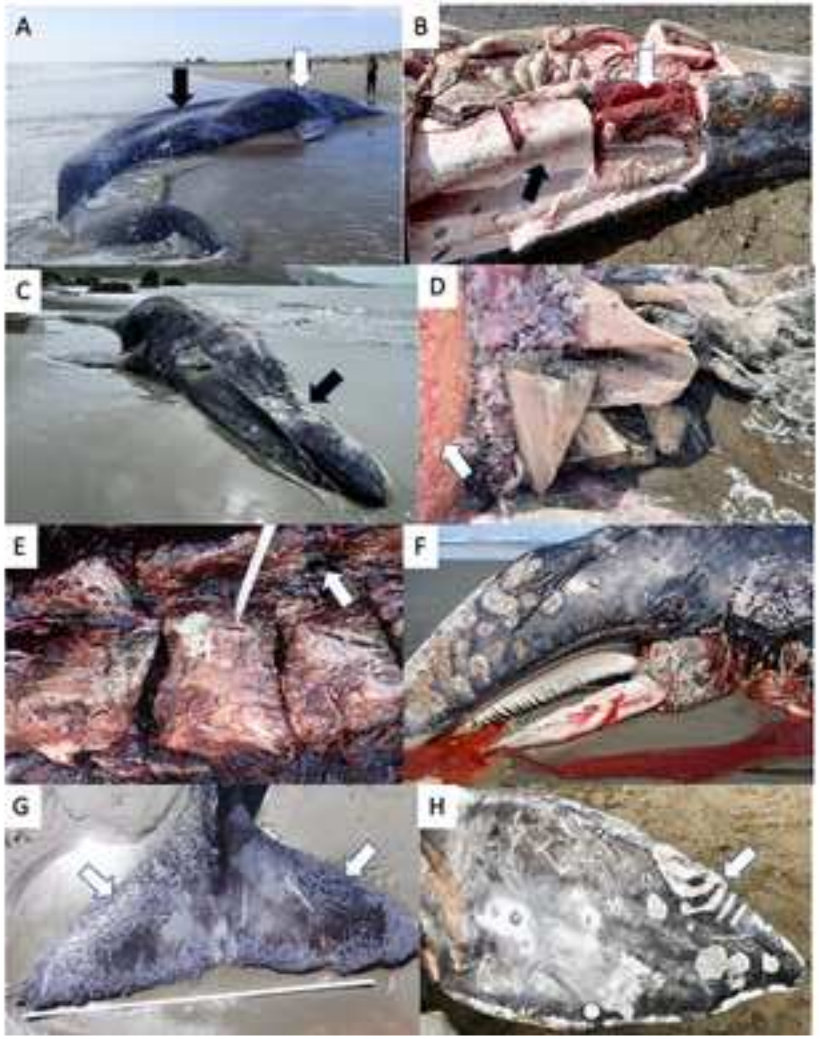

Click here to access/download;Figure;Fig3.tiff.tif

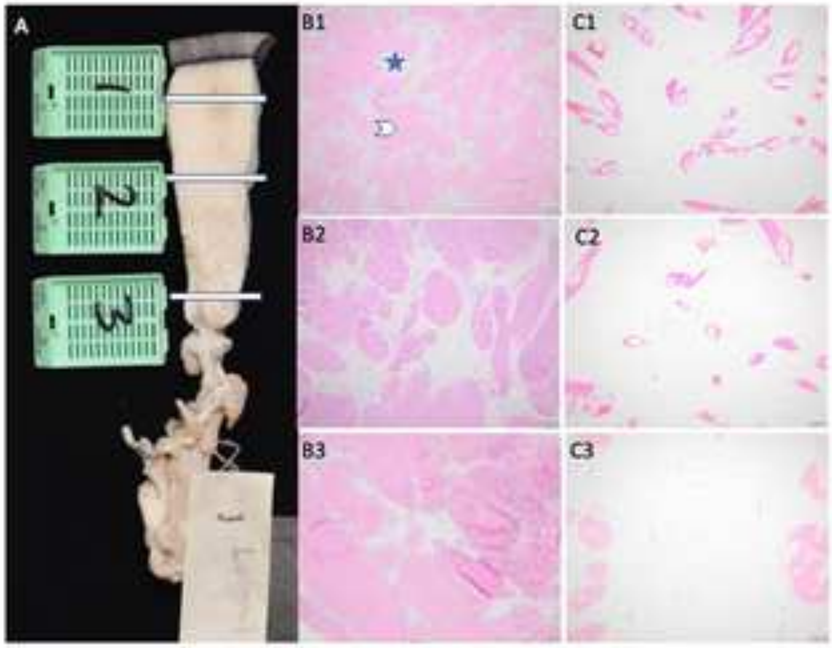

Click here to access/download;Figure;Fig4.tiff.tif

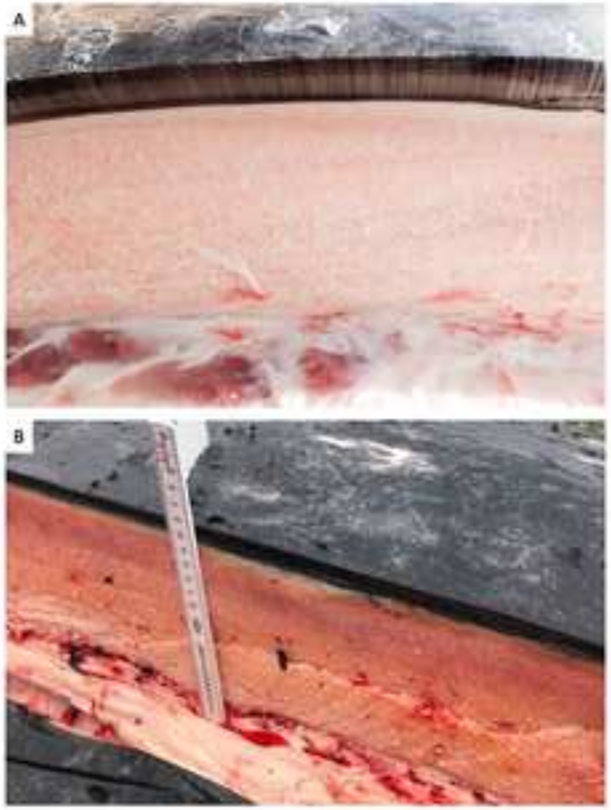

 Click here to access/download;Figure;Fig5.tiff.tif

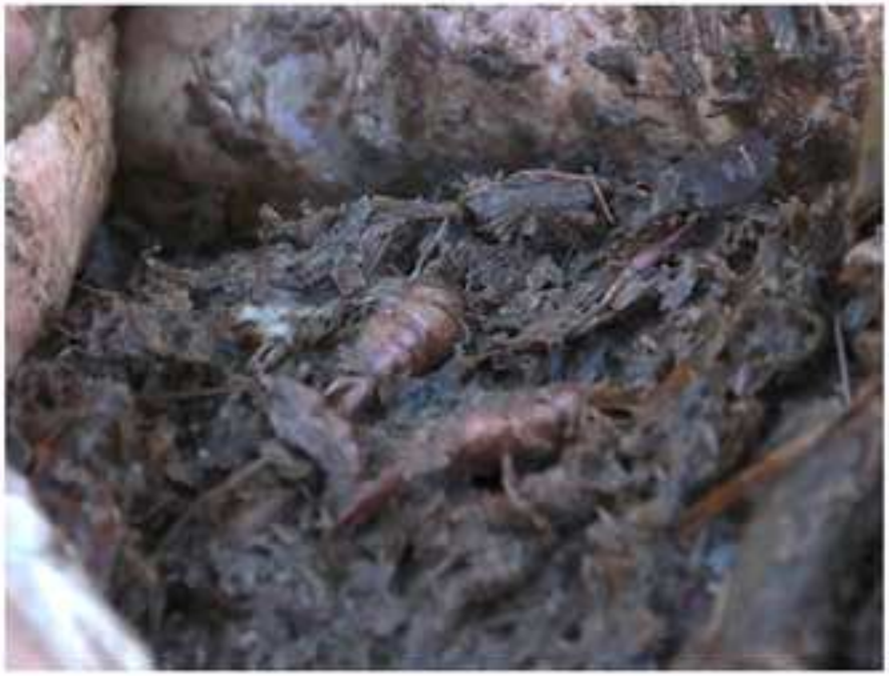

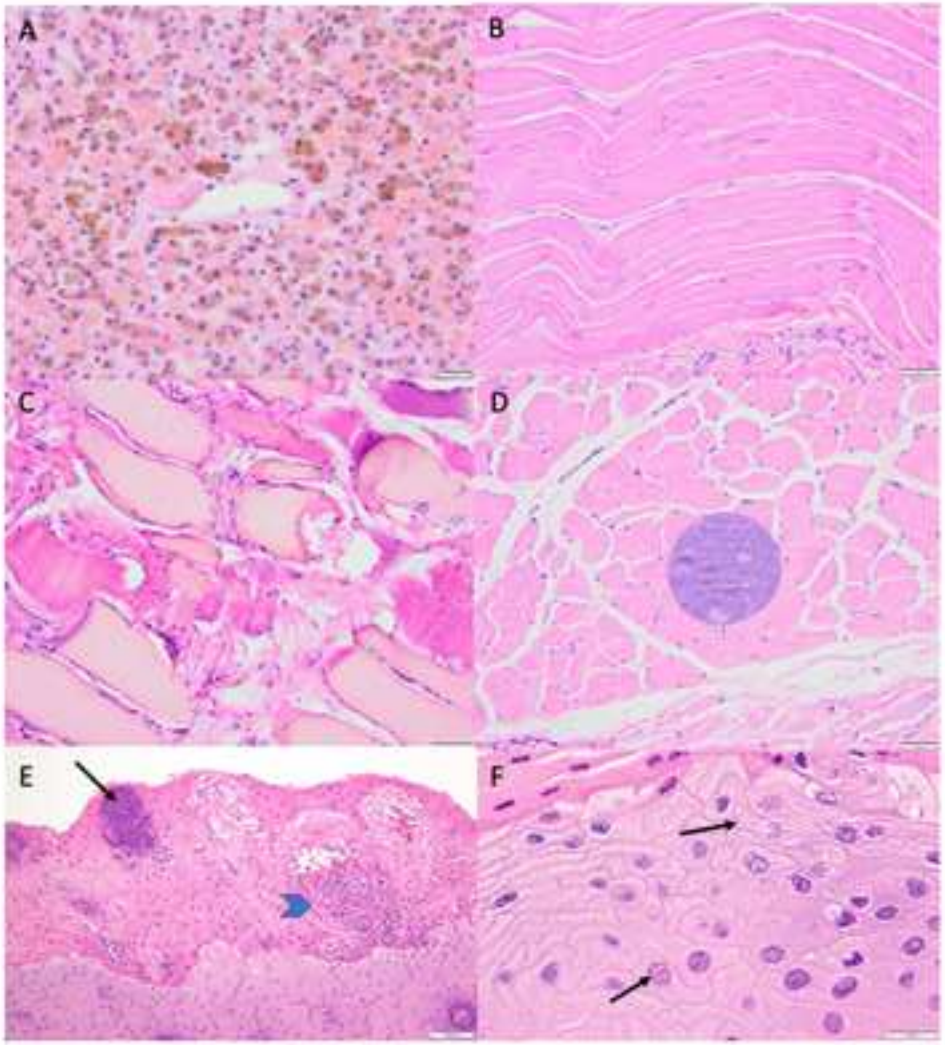
A
B
C
D
E
F

Figure

Click here to access/download;Figure;Fig7.tiff.tif

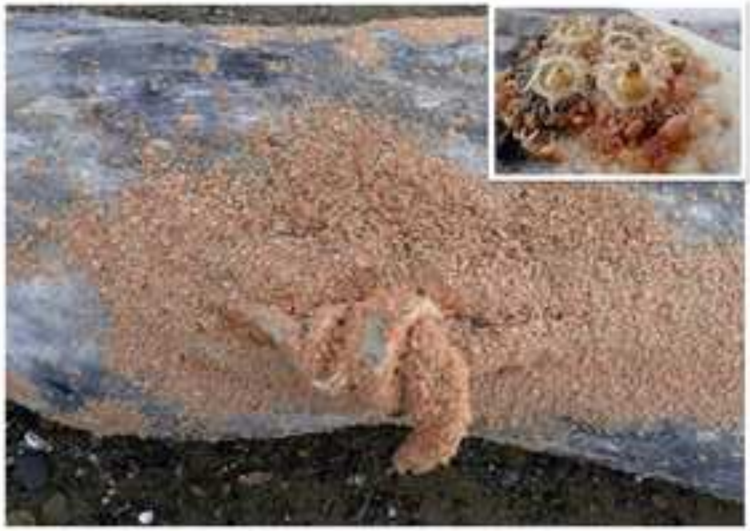

Click here to access/download
Supporting Information
S1 File. Gray Whale Nutritional Condition.docx

Click here to access/download
Supporting Information
S2 File. Killer Whale Predation.docx
